# The effects of intelligibility on conflict resolution and monitoring during speech recognition in noise

**DOI:** 10.3758/s13414-026-03271-2

**Published:** 2026-05-06

**Authors:** Susan Teubner-Rhodes, Anna Pusser, Rebecca Dunterman

**Affiliations:** Department of Psychological Sciences, Auburn University, 226 Thach Hall, Auburn, AL 36849 USA

**Keywords:** Cognitive and attentional control, Speech perception, Spoken word recognition

## Abstract

Listeners have difficulty understanding speech in noisy environments, confusing similar sounding words. Cognitive control may be important for segregating the speech signal from background noise and/or selecting between phonological competitors in the mental lexicon. Although cognitive control abilities may be related to speech recognition in noise, the extent to which engaging cognitive control causally improves speech recognition in background noise is unclear. The present study presented pictures that matched (congruent trials) or were phonological neighbors (incongruent trials) of the spoken word to manipulate cognitive control during speech recognition in multitalker babble at + 6 and + 8 dB signal-to-noise ratios (SNRs). As conflict increases cognitive control levels, performance following incongruent trials indexes the effect of cognitive control on recognizing speech in noise. Following conflict, participants became faster and more accurate at repeating back speech at + 6 dB SNR and more accurate at + 8 dB SNR. When comparing across SNRs (including a + 4 dB SNR from a previous study), higher SNRs reduced the interference effect, predicting better recognition on incongruent trials; however, SNR did not significantly modulate postconflict improvements in word recognition. Results suggest engaging cognitive control improves speech recognition in noise across SNR levels where recognition is challenging but possible.

**Supplementary Information:**

The online version contains supplementary material available at 10.3758/s13414-026-03271-2.

Understanding speech in noisy environments is challenging. Background noise can reduce audibility of the speech signal and create confusion between sound sources (e.g., Grant & Walden, 2013; Summers & Molis, 2004). Additionally, individual differences in age (Dubno et al., 1984; McCreery et al., 2020), verbal ability (McCreery et al., 2020; Nuesse et al., 2018), and working memory (Akeroyd, 2008; Besser et al., 2013; McCreery et al., 2020) contribute to performance even among normal hearing listeners. Increased difficulty understanding speech in noise could reflect the cognitive demands necessary to focus attention on the speech signal, ignore irrelevant background noise, and/or select between similar sounding words in the mental lexicon. Cognitive control, the ability to direct attention and resolve conflict between competing sources of information, may be important for recognizing speech in background noise. Indeed, individuals with higher cognitive control have better spoken word recognition for target words that have many phonological neighbors (Sommers & Danielson, 1999; Taler et al., 2010). This suggests that cognitive control resolves phonological conflict, which occurs when similar sounding lexical items are simultaneously activated as potential matches for the input, by helping listeners increase activation of a target word and/or suppress phonologically similar lexical items. However, it is unclear the extent to which engaging cognitive control directly benefits speech understanding in noisy environments. The present study assesses whether inducing cognitive control through stimulus conflict improves speech recognition in noise and how speech intelligibility, manipulated by the signal-to-noise ratio (SNR), affects the role of cognitive control during speech recognition in noise.

## Cognitive demands of speech recognition in noisy environments

Evidence suggests that listeners activate many, phonologically similar words in the mental lexicon during spoken word recognition (e.g., Allopenna et al., 1998; Dirks et al., 2001). When a speaker references an object in a display, listeners initially move their eyes to both the referenced object and a competitor object with the same phonological onset (Allopenna et al., 1998). Looks to the referenced object begin to exceed looks to the onset competitor around 400 ms after word onset (Allopenna et al., 1998). Listeners also exhibit more looks to a rhyming competitor object than objects that are not phonologically related to the speech stimulus, but these occur later in the recognition period from about 300–700 ms (Allopenna et al., 1998). Spoken word recognition accuracy is higher for words that are phonologically and lexically more likely than their neighbors, especially at intermediate levels of background noise (Luce & Pisoni, 1998). Dirks et al. (2001) found that, in both quiet and noisy conditions, listeners had better recognition for words with few or infrequent phonological neighbors compared with words with many frequently occurring neighbors. That is, a word that overlaps with many similar sounding neighbors in the mental lexicon is less likely to be recognized, especially when the neighbors occur more often than the target. Taken together, the results of eye-tracking and offline speech recognition studies support continuous mapping models of word recognition (e.g., McClelland & Elman, 1986), which suggest that the listener simultaneously activates many words that are partial matches for the speech signal as it unfolds, until the best match wins out.

Background noise can interfere with the speech signal, leading to uncertainty about one or more phonemes and decreasing the likelihood of correct word recognition. As SNR decreases, word recognition follows a sigmoidal psychometric function of decreasing accuracy until speech becomes fully unintelligible (e.g., Wu et al., 2016). Background noise can disrupt speech recognition through 1) energetic masking, when the acoustic energy of the noise dominates the speech signal or 2) informational masking, when the acoustic or semantic similarity of the background noise leads to confusability with the speech signal (Wilson et al., 2012). According to Mattys et al. (2012), energetic masking primarily requires separating the speech signal from noise and engaging selective attention for correct recognition, while informational masking requires resolving semantic interference and overcoming cognitive load. Background noise has been associated with increased listening effort across several different metrics. Task-evoked increases in pupil dilation can index locus coeruleus activity that increases attentional focus during cognitively demanding tasks (Gilzenrat et al., 2010). During speech recognition in noise, the pupil dilates as the SNR decreases (Kramer et al., 1997; McMahon et al., 2016; Zekveld et al., 2010, 2018), suggesting a commensurate increase in listening effort (see Teubner-Rhodes & Kuchinsky, 2020, for review), although dilation decreases again for SNRs with very poor intelligibility (e.g., correct recognition less than 20–40%; Ohlenforst et al., 2017, 2018). Participants have higher self-reported effort ratings when recognizing speech in noise than in quiet (Picou et al., 2011). They also perform more poorly on a secondary task when listening in noise than in quiet (Picou & Ricketts, 2014) and as SNR decreases (Wu et al., 2016). Thus, background noise not only decreases word recognition performance but also appears to make listeners work harder to try to understand speech, as long as correct recognition is possible.

Cognitive control allows individuals to prioritize goal-relevant information in the face of competing sources of information (Mackie et al., 2013), and it may play an important role in understanding speech in noisy environments. The listener might employ cognitive control to enhance speech recognition by 1) increasing attention to the speech signal while ignoring irrelevant background noise and/or 2) selecting the target word in the mental lexicon while inhibiting lexical competitors. The former is supported by evidence that semantic priming from an unattended speech signal is reduced in individuals with better response inhibition on an antisaccade task (Perrone-Bertolotti et al., 2017). Additionally, Stenbäck and colleagues (2021, 2022) showed that those with better response suppression on the Swedish Hayling task, which requires producing an unexpected word to complete a highly predictable sentence, had higher recognition of speech presented in maskers consisting of a single or multiple talkers but not in maskers constructed from speech-shaped noise. Taken together, this evidence suggests that cognitive control may help listeners suppress attention to background noise, especially when it is acoustically or semantically confusable with the speech signal.

There is also evidence that cognitive control may help listeners decide between competitors in the mental lexicon. Higher cognitive control ability was associated with better recognition of words presented in speech-shaped noise in both predictable and unpredictable sentence contexts, but only for lexically difficult words with many frequent neighbors; cognitive control ability was not related to recognition of words with fewer neighbors (Sommers & Danielson, 1999). Individuals with better cognitive control as measured by the color-word Stroop test also exhibited smaller neighborhood density effects on word recognition accuracy in a − 3 dB SNR condition but not a higher + 10 dB SNR condition (Taler et al., 2010). Cognitive control thus appears to be related to recognition of words when individuals have to select a single target among many phonological neighbors, especially as the speech signal becomes less discriminable from background noise. The mechanism by which cognitive control might select target words is unclear, but it may enhance activation of lexical representations that best match the acoustic input and/or suppress activation of lexical representations that are relatively weaker matches.

## Conflict monitoring during speech recognition in noise

Most existing evidence for the role of cognitive control in understanding speech in noise relies on correlations between cognitive control ability and speech recognition accuracy (e.g., Sommers & Danielson, 1999; Taler et al., 2010). While suggestive, such correlations cannot show that using cognitive control *causally* benefits speech recognition in background noise. To evaluate the causal role of cognitive control during speech recognition in noise, we manipulate conflict from trial-to-trial and assess conflict monitoring. Conflict monitoring is the process of detecting conflict and upregulating cognitive control (Botvinick et al., 2001). It is characterized by faster and/or more accurate responding following stimulus conflict on cognitive control tasks (e.g., Akçay & Hazeltine, 2011; Egner & Hirsch, 2005; Gratton et al., 1992; Jiménez & Méndez, 2013; Notebaert & Verguts, 2008). Such adaptive performance adjustments are known as congruency sequence effects, which occur when the difference in performance between congruent and incongruent trials is reduced after a preceding incongruent trial (Duthoo et al., 2014; Egner, 2007). Conflict monitoring theory attributes congruency sequence effects to the time it takes to notice conflict and reactively increase engagement of cognitive control (Botvinick et al., 2001). That is, when participants first encounter conflict, their engagement of cognitive control is low. Detecting conflict triggers an increase in activation of cognitive control resources, but this process takes time, resulting in relatively slow, low-accuracy responses. Following conflict, however, cognitive control engagement is high, and participants are prepared to resolve a subsequent conflict quickly and accurately. Conflict monitoring theory is supported by neurological evidence that conflict detection increases activity in the dorsal anterior cingulate cortex (dACC), which predicts subsequent increases in lateral prefrontal activity and performance improvements (Kerns, 2006; Kerns et al., 2004). Interestingly, a similar brain network including dACC and the lateral prefrontal cortex is engaged when listening to low-intelligibility speech, suggesting that recognizing degraded speech may tap into general cognitive control resources (Eckert et al., 2016).

We recently developed a novel picture–speech recognition in noise task (SPRiNT) to evaluate if manipulating cognitive control engagement from moment-to-moment directly affects performance during speech recognition in noise (Teubner-Rhodes et al., 2024). The SPRiNT presents a series of spoken words in continuous multitalker babble and asks participants to repeat each word that they hear. Each word is presented along with a picture, which is the same as the spoken word on congruent trials (e.g., *pipe*– “pipe”) and is a phonological neighbor of the spoken word on incongruent trials (e.g., *chair*– “dare”). According to conflict monitoring theory, the conflict engendered by the competing picture and spoken word on incongruent trials should increase recruitment of cognitive control resources. If cognitive control causally benefits speech recognition in noisy environments, then word recognition should improve after incongruent trials. This is exactly what we found: participants were significantly more accurate (+ 17% points) at identifying spoken words on incongruent trials that followed an incongruent trial than those that followed a congruent trial (Teubner-Rhodes et al., 2024). These findings demonstrate conflict monitoring during speech recognition in noise, supporting a causal role for cognitive control in recognizing spoken words when phonological conflict arises.

Applying conflict monitoring theory (Botvinick et al., 2001) to speech recognition in noise is compatible with the Ease of Language Understanding model (ELU; Rönnberg et al., 2013, 2019, 2021), which explains how working memory facilitates speech recognition in degraded conditions. According to the ELU, when the speech signal is clear, bottom-up linguistic input is subject to a process of Rapid, Automatic, Multimodal Binding of Phonology (RAMBPHO), with the resulting phonological representation compared with stored lexical representations in semantic memory. When the match between the RAMBPHO and the stored lexical representations is insufficient to support successful recognition, then working memory resources are used to effortfully recover the intended meaning. This postdiction process of working memory-mediated repair is followed by prediction, in which context primes relevant input (Rönnberg et al., 2019, 2021). This cycle of postdiction and prediction is analogous to conflict monitoring, where conflict triggers cognitive control resources to select between incompatible mental representations/responses and tune attention to task-relevant information on subsequent trials (Botvinick et al., 2001). Although ELU emphasizes activation of working memory, cognitive control is a component of working memory that directs attention and inhibits irrelevant information (Baddeley, 2000). In other words, cognitive control works together with storage and processing units to govern the contents of working memory. Upregulating cognitive control in response to phonological conflict to benefit word recognition is consistent with ELU; however, ELU focuses on interpreting input within a broader sentence context, while conflict monitoring theory proposes that cognitive control remains elevated to support subsequent recognition even of isolated words in noise.

## The present study

Cognitive control may play a greater role in understanding speech in noise at lower SNRs, due to the increased demands of segregating the auditory signal from noise and greater stimulus uncertainty from reduced activation of the target word relative to phonological neighbors. Our prior SPRiNT study did not investigate the effect of SNR on cognitive-control-driven improvements in word recognition, as words were presented at a fixed + 4 dB SNR (Teubner-Rhodes et al., 2024). The present study aims to 1) extend our prior findings that conflict improves subsequent speech recognition in noise to different SNR conditions and 2) understand how SNR affects the role of cognitive control during speech recognition in noise.

To achieve these aims, we conduct a new experiment investigating the effect of conflict on current and subsequent word recognition in 12-talker babble (Kalikow et al., 1977) at + 6 dB and + 8 dB SNR and compare the magnitude of postconflict improvements across intelligibility levels. We selected these higher SNRs because mean accuracy levels for the same speech and babble stimuli at + 4 dB SNR (Teubner-Rhodes et al., 2024) were 39% (range: 17–63%) and 57% (range: 0–83%) for incongruent trials preceded by congruent and incongruent trials, respectively; we expected + 6 and + 8 dB SNRs to require cognitive control without approaching floor or ceiling performance. Previous research using the same multitalker babble masker in the absence of pictures at + 3 and + 10 dB SNRs observed mean accuracy of 66% and 91% in normal hearing young adults (Vaden et al., 2013) and 43% and 70% in normal hearing older adults (Vaden et al., 2015), respectively. This suggested that our selected SNRs would be in a range where speech recognition would be possible but still elicit speech recognition errors.

We developed three competing hypotheses for how SNR may affect the role of cognitive control during speech recognition in noise, measured by postconflict improvements in word recognition. 1) If cognitive control primarily selects between similar-sounding words in the mental lexicon, then postconflict improvements should not be affected by SNR. Because the phonological conflict between the picture and the spoken word is present across SNRs, it will give rise to competition between activated words in the mental lexicon and postconflict improvements regardless of SNR. 2) If cognitive control primarily increases attention to the target speech and suppresses background noise, then postconflict improvements in word recognition should increase as SNR decreases. As speech becomes less intelligible, the benefit from engaging cognitive control increases. Decreasing SNR increases uncertainty about phonemes in the target word, leading to reduced activation of the target that makes it more difficult to discriminate. Lower SNRs may therefore require increased cognitive control to help segregate the target speech signal from interfering background noise. 3) Finally, cognitive control may have a negative, quadratic effect on speech recognition in noise. While the need for cognitive control increases as SNR decreases, the effort of applying cognitive control outweighs the benefit when intelligibility becomes quite poor (Eckert et al., 2016). Such inverted U-shaped relationships have been observed between physiological indices of effort—including activation of the cingulo-opercular system (Wild et al., 2012; Zekveld et al., 2006) and pupil dilation (Ohlenforst et al., 2017, 2018)—and word recognition in degraded listening conditions. To test the hypotheses related to SNR effects, we compared data collected at + 6 and + 8 dB SNR to the + 4 dB SNR collected in an earlier study (Teubner-Rhodes et al., 2024).

## Method

### Participants

One-hundred and twenty-nine participants were recruited from the Auburn, AL-area using flyers, word of mouth, and the Auburn Department of Psychology Sona Systems participant pool. Sixty-nine were excluded for the following reasons: technical difficulties during the SPRiNT (+ 6 dB: *n* = 3; + 8 dB: *n* = 3), dropping out prior to study completion (+ 6 dB: *n* = 9; + 8 dB: *n* = 11), or ineligibility (+ 6 dB: *n* = 16; + 8 dB: *n* = 27) due to neurological or psychological disorders, a history of seizures, > 25 dB of hearing loss from 500 to 4000 Hz in either ear or > 20 dB asymmetry between the ears, color blindness, corrected visual acuity worse than 20/20, or having risk factors for severe illness from COVID-19. Dropouts were typically due to participants not scheduling or not showing up to the in-person lab visit after the initial online consent and screening.

The final sample included 60 healthy, young adult monolingual native speakers of American English between the ages of 18 and 39 years. Thirty participants completed the + 6 dB and 30 completed the + 8 dB SNR condition. Participant demographics for each condition are reported in Table 1; the groups were not significantly different on any of the characteristics measured.

**Table 1:** Participant Demographics, Hearing, and Naming Scores for the + 6 and + 8 dB SNR Conditions

	+ 6 dB SNR		+ 8 dB SNR		+ 6 vs. + 8 dB SNR	
	*n*	%	*n*	%	χ^2^(1)	*p*
*Gender*					3.07	0.08
Women	25	83%	19	63%		
Men	5	17%	11	37%		
*Ethnicity*					0.11	0.74
White, non-Hispanic	24	80%	25	83%		
Other^1^	6	20%	5	17%		
	*M*	*SD*	*M*	*SD*	*t(58)*	*p*
Age (in years)^2^	18.79	1.11	19.90	3.81	−1.50	0.14
Education	12.93	1.28	13.20	1.34	−0.79	0.44
EHI	87.17	38.72	77.19	54.98	0.81	0.42
Maternal Education	16.30	1.86	16.47	1.91	−0.34	0.73
Paternal Education	16.00	2.84	16.83	2.26	−1.26	0.21
PTA^3^	10.13	0.51	10.47	1.03	−1.62	0.11
Picture Naming: 1st	0.92	0.03	0.93	0.03	−1.05	0.30
Picture Naming: 2nd	0.98	0.02	0.98	0.01	−0.79	0.43

*EHI * Edinburgh Handedness Inventory laterality quotient; *PTA* pure-tone average calculated as the mean of pure-tone thresholds (in dB hearing loss) at 500, 1000, 2000, and 4000 Hz in the better ear; Education is measured in years completed, with a maximum possible value of 20 for doctoral degrees. Picture naming is measured as the proportion correct picture naming accuracy on the 1st and 2nd attempts

^1^Other ethnicities in the + 6 dB SNR included 2 Hispanic White, 1 Asian, 1 Native Hawaiian or Pacific Islander, and 2 multiple ethnicities. Other ethnicities in the + 8 dB SNR included 3 Black, 1 Hispanic White, and 1 Asian

^2^Age was not reported for one participant in the + 6 dB SNR

^3^PTA results were not saved after confirming eligibility for one participant in the + 6 and one in the + 8 dB SNR condition

We used the *simr* package (Green & MacLeod, 2016) to estimate the power from a previously collected dataset, where the interaction between preceding and current trial type had an effect size of .80 on word recognition accuracy (Teubner-Rhodes et al., 2024). Using our study design of 24 trials per condition per participant and a reduced effect size of .75 (Kumle et al., 2021), power simulations showed that we had 100% power to detect a significant interaction (at α < .05) between preceding and current trial type in our full sample (*n* = 60) and 70% power to detect this interaction within each SNR (*n* = 30). We note that, because the current study uses the same design as the previous work, which showed a significant preceding-by-current trial type interaction, the resulting power will necessarily be high. The simulations were conducted with a logistic mixed-effects model that included the random intercept by-subjects and random slopes for trial type, with the same intraclass correlation coefficient as the model of the original data (ICC = 0.035)—we were unable to simulate models with a more complex random effects structure as these did not converge. These power estimates are limited to the analyses of congruency sequence effects, which are reflected in the two-way interaction between preceding and current trial type. No a priori estimate of the effect size for the three-way interaction between preceding trial, current trial, and SNR was available because the present study is the first to investigate the effect of SNR on congruency sequence effects, to the best of our knowledge.

Two participants in the + 8 dB SNR condition provided ambiguous data on their language background. One reported that they were a native speaker of American English who did not speak another language fluently, but they also reported having lived in countries where English was not the primary language spoken during early childhood. The other reported that they were a native speaker of American English who had never lived outside the United States, but they also reported that they spoke a second language fluently, despite not answering questions asking them to specify the second language and their age of acquisition. We opted to include these participants in the final sample, as they were native speakers of American English and therefore should be able to recognize the words in the experiment.

All procedures were conducted and informed consent obtained in accordance with the Declaration of Helsinki and the approved Auburn University IRB protocol. Participants received their choice of 1 Sona credit toward coursework or $10 per hour for their participation.

### Materials

The stimuli for the SPRiNT are described in full by Teubner-Rhodes et al. (2024). Briefly, stimuli were 150 picture–speech pairs that were matching on congruent trials (*n* = 60), were phonological neighbors on incongruent trials (*n* = 60), or were unrelated on filler trials (*n* = 30). The purpose of presenting pictures that sounded like the spoken word on incongruent trials was to create conflict between the target word and its phonological neighbor in the mental lexicon. Filler trials blocked the expectation that all words sounded like their pictures to keep participants from generating responses based on the picture alone.

Spoken words were recordings of a woman native speaker of American English (author S.T.R.) that were less than 1 s in duration. All words ranged from two to five phonemes in length. Pictures were color photographs of isolated objects with name agreement of 50% or higher from the Bank of Standardized Stimuli (Brodeur et al., 2010, 2014). Words and picture names were matched for number of phonemes, neighborhood density, and frequency across congruency conditions, and pictures were additionally matched for name agreement.

### Procedure

We largely followed the procedure described in Teubner-Rhodes et al. (2024), except that speech stimuli were presented at a + 6 dB or + 8 dB SNR depending on the condition. Participants first completed a series of Qualtrics questionnaires asking questions related to study exclusionary criteria, language learning and attention history, demographic information, and handedness during a 15-min Zoom call. Participants who were eligible came to the lab, where they completed a vision screening and a hearing test to ensure clinically normal vision (20/20) and pure-tone thresholds. Pure-tone thresholds were tested in both ears at conventional frequencies from 250–8000 Hz via Shoebox Standard Audiometry iPad app in accordance with ANSI 3.6 audiometer standards (American National Standards Institute, 2010).

Participants then completed the SPRiNT, which presented a series of picture–word pairs in multitalker babble. Multitalker babble provides an ecologically valid simulation of real-world background noise. As a masker, it is acoustically similar to target speech and is hypothesized to increase the cognitive demand of listening relative to modulated and unmodulated noise maskers (Dryden et al., 2017). A background babble track consisting of 12 simultaneous talkers (Kalikow et al., 1977) was presented continuously at 60 dB SPL while the spoken words were presented at 66 or 68 dB SPL to achieve a + 6 or + 8 dB SNR, respectively. With 12 simultaneous talkers, masking is expected to be primarily energetic rather than informational in nature (Carhart et al., 1975; Freyman et al., 2004; Wilson et al., 2012).

Participants were instructed that they would hear a series of words presented in background noise and asked to repeat each spoken word or say “nope” if they did not understand it. They were informed that they had to respond during the cued response period or else their response would not be recorded. They were also told that they would see pictures, and they should look at each picture, but their task was to repeat the spoken word. Each trial included a black fixation cross for 1.0 s, a picture for 1.5 s, a spoken word beginning 500 ms after picture onset and lasting < 1.0 s, and a red fixation cross for 2.0 s that cued the response period.

**Fig. 1:**
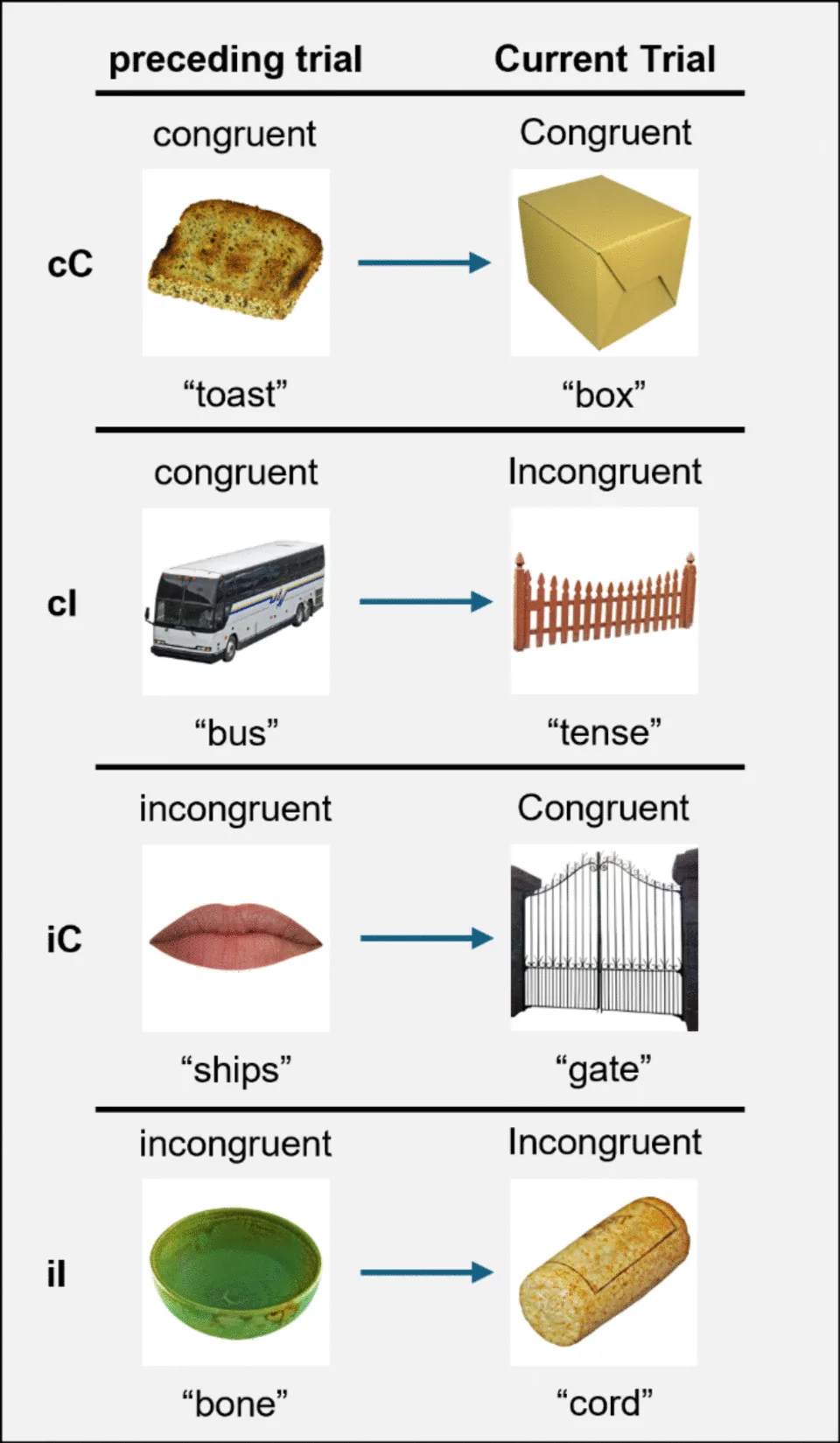
Illustration of critical trial sequences: cC, cI, iC, and iI. *Note*. From left to right in each row, the corresponding picture names are toast, box, bus, fence, lips, gate, bowl, and cork. Pictures (Brodeur et al., 2010, 2014) are presented for illustration only and are not to scale

Participants wore Sennheiser HDA 300 over-the-ear headphones and sat at a computer equipped with E-Prime 3.0 software (Psychology Software Tools, 2016) to present the experiment and record participants’ responses. Participants completed 20 practice trials before proceeding to the main experiment, consisting of 150 trials in a pseudorandomized order, such that there were 96 critical trial sequences: 24 congruent-congruent (cC), 24 congruent-incongruent (cI), 24 incongruent-congruent (iC), and 24 incongruent-incongruent (iI; see Fig. 1). Each trial presented a unique stimulus; thus, there were no stimulus repetitions or stimulus–response contingencies that can sometimes artificially inflate the magnitude of congruency sequence effects (Schmidt, 2013, 2019). Participants were offered a brief break after 75 trials before completing the remaining 75. Both blocks began with a filler trial so that the first and second trials of each block were not critical trials. The entire SPRiNT, including instructions and practice trials, took 15–20 min to complete.

Finally, participants completed a picture-naming task to evaluate if the pictures in the SPRiNT evoked the intended word. Participants advanced through a Microsoft PowerPoint presentation that displayed each of the 150 pictures from the SPRiNT one at a time. They were instructed to say the first word that came to mind for each picture, then wait for the researcher. The researcher asked them to give a second name if their first response was not the expected one before telling participants to move onto the next picture. Naming accuracy was initially above 90% and increased to near ceiling by the second attempt in both the + 6 and + 8 dB SNR conditions, and the groups did not significantly differ in their naming accuracy on either attempt (see Table 1). This suggests that the pictures generally activated the intended word for participants who completed the SPRiNT in both the + 6 and + 8 dB SNRs.

### Scoring

Participants’ SPRiNT responses were scored by two independent raters, with at least one listening via an intercom in a separate lab room and another checking the digital audio recordings after the session. Only responses that repeated the word exactly, with no additions, omissions, or substitutions, were marked as correct. Any disagreements were discussed and resolved with a third rater. The raters agreed on the response accuracy for 96% of + 6 dB trials (Cohen’s *κ* = 0.90) and 95% of + 8 dB SNR trials (Cohen’s* κ* = 0.87). Responses that were missing or unintelligible were excluded from analyses (+ 6: *n* = 8, 0.2% of the data; + 8: *n* = 7, 0.2% of the data).

The speech onset time (SOT) was established for each trial via an algorithm designed to find the onset of each word in R (Vaden et al., 2022). The program produced a spectrogram of each spoken response and added a line to indicate the point that the algorithm determined the SOT to be. The automated SOTs were then verified by two raters. Using Audacity, the raters listened for the SOT and manually corrected them when misplaced. The two SOT ratings were averaged together, unless they differed by more than 40 ms, in which case an expert rater (author S.T.R.) determined the correct SOT using Praat. For the + 6 dB SNR condition, raters had strong absolute agreement with an average-measure ICC(2,2) of 0.984, 95% CI [0.983, 0.985; *F*(4447, 3443) = 63.2, *p* < 0.001. The corrected SOTs deviated from the auto-generated times on 31% of trials (24% by more than 40 ms). For the + 8 dB SNR condition, raters also had strong absolute agreement with an average measure ICC(2,2) of 0.993, 95% CI [0.992, 0.993]; *F*(4449, 4362) = 141, *p* < 0.001, and the corrected SOTs deviated from the auto-generated times on 25% of trials (16% by more than 40 ms).

### Analyses

We conducted two sets of regression analyses to test our hypotheses: 1) we examined congruency sequence effects at + 6 and + 8 dB SNR to evaluate whether cognitive control benefits speech recognition at each noise level, and 2) we used additional data at + 4 dB from a prior study (Teubner-Rhodes et al., 2024) to evaluate the extent to which SNR modulated congruency sequence effects to determine whether SNR impacts the role of cognitive control during speech recognition in noise. Each of these analyses is described in detail below, but we first provide an overview of our analytical approach.

All analyses were conducted in R (Version 4.1.1). We conducted mixed-effects regression models using preceding trial type, current trial type, and their interaction as fixed effects, using a dummy coding scheme with congruent as the reference level for both preceding and current trial type. Congruency sequence effects are indexed by a significant interaction between preceding and current trial type, particularly when driven by better performance (i.e., higher word recognition accuracy and/or faster responding) on iI than cI trials. Such postconflict improvements are thought to demonstrate reactive engagement of cognitive control (Botvinick et al., 2001) and would demonstrate that cognitive control benefits word recognition in background noise.

Only critical trials (cC, cI, iC, iI) that followed correct responses were included in analyses. This approach is considered best practice when examining congruency sequence effects (Braem et al., 2019), as performance on trials following incorrect responses could reflect posterror rather than postconflict effects. Analyses for SOTs were restricted to correct trials only (in addition to removing posterror trials), as SOTs for incorrect trials do not reflect the time needed to successfully recognize the word. Because excluding trials following errors results in data loss, especially for iC and iI conditions that follow incongruent trials, we conducted secondary analyses that included posterror trials to ensure that this procedure did not affect the results. Results of analyses including posterror trials are reported in the Supplementary Materials, and any discrepancies are noted in the main text.

We tested the full model justified by the study design, as is recommended for mixed-effects models (Barr et al., 2013). This included random slopes by subject but did not include random effects of items, as items were not sampled randomly but fixed to match lexical characteristics across conditions. As conducting model comparisons for the SNR analyses required using the same random effects term across models, we used only the random intercept during model selection and then added random slopes when estimating parameters of the best-fitting model. If any model failed to converge, we simplified the random effects term by first removing the interaction and then main effects, one at a time, until the model converged. Adjusted intraclass correlation coefficients for the random effects of all final models are reported using the “icc” function from the performance package (Lüdecke et al., 2021).

Inspection of conditional residuals for SOTs for both analyses revealed that their distribution had a slight positive skew. Neither log-transforming the SOTs nor removing influential observations with a high Cook’s distance relative to other points changed the inference of model fixed effects. We report raw SOTs in the main text for ease of interpretation and include the results for transformed SOTs in the Supplementary Materials.

To verify that effects were not an artifact of temporal dependencies, we tested models both with and without normalized trial number included as a fixed effect. While accuracy on cC trials decreased over time and accuracy on cI trials increased over time, trial number did not modulate congruency sequence or SNR effects that were the focus of the present study. For SOTs, participants became faster on both cC and iI trials over the course of the task, but a postconflict advantage for incongruent trials was evident throughout. Congruency sequence and SNR effects were comparable whether or not trial number was included in the model. Results including normalized trial number are reported in the Supplementary Materials.

#### Analyses of congruency sequence effects

Analyses of congruency sequence effects assessed whether significant postconflict improvements in word recognition replicated at + 6 and + 8 dB SNR. We used the “glmer” function in the *lme4* package (Version 1.1—29) to analyze correct or incorrect word recognition on each trial across both SNRs with mixed-effects logistic regression fit by maximum likelihood using Laplace approximation, which is standard practice for models of this type. We analyzed SOTs for correct word recognition trials with mixed-effects linear regression fit by restricted maximum likelihood (REML) using the “lmer” function in *lmerTest* package (Version 3.1.3). For linear mixed-effects models, restricted maximum likelihood is considered more suitable than maximum likelihood as it produces more reliable estimates (Luke, 2017). We approximated *p* values using Satterthwaite’s degrees of freedom method, which effectively controls type I error rates (Luke, 2017).

The tested models for word recognition (REC) and SOTs are listed below, where PREC is preceding trial type, CURR is current trial type, SNR is the mean-centered SNR, and SUB is the by-subjects random effects term. Simplified interaction terms are shown for readability, but all pertinent main effects were also included:

- Model 1: REC = PREC × CURR × SNR + (1 + PREC × CURR | SUB).
- Model 2: SOT = PREC × CURR × SNR + (1 + PREC × CURR | SUB).

In both cases, the model with the full random effects structure failed to converge, and so random effects were systematically simplified until convergence was reached.

To further understand congruency sequence effects, we used the *emmeans* package (Version 1.11.1) to estimate the interaction effect at each SNR and conduct SNR-conditional pairwise comparisons between cC, cI, iC, and iI trials using the Tukey adjustment for multiple comparisons. The contrast of cC versus iC and cI versus iI examined the effect of preceding trial congruency when the current trial was congruent or incongruent, respectively. Larger postconflict improvements for current incongruent trials (cI versus iI) compared with current congruent trials (cC versus iC) would support cognitive control-based adjustments in performance.

#### Analyses of SNR effects

Analyses of SNR effects included + 4 dB SNR data reported previously (Teubner-Rhodes et al., 2024) as well as the + 6 and + 8 dB data reported here. We compared models without the SNR term (model 3) and with orthogonal linear (model 4) and quadratic (model 5) SNR terms and their interactions with preceding and current trial type to evaluate how SNR modulates congruency sequence effects in spoken word recognition accuracy and SOTs (collectively referred to as DV in the models below). The SNR terms were centered and scaled to be orthogonal using the poly function. Model 3 tests the hypothesis that SNR does not modulate cognitive control effects, model 4 tests the hypothesis that decreasing SNR increases cognitive control effects, and model 5 tests the hypothesis that decreasing SNR increases cognitive control effects up to a point, until performance becomes too low. We compared models using the likelihood ratio test to identify the best fit to the data and report the results for the best-fitting model.

- Model 3: DV = PREC × CURR + (1 | SUB)
- Model 4: DV = PREC × CURR × SNR + (1 | SUB)
- Model 5: DV = PREC × CURR × SNR × SNR^2^ + (1 | SUB)

In order to sensibly compare models with different fixed effects via the likelihood ratio test, it was necessary to fit them using maximum likelihood and hold the random effects term constant (i.e., using the by-subjects random intercept only). However, we re-fit the selected model using best practices—namely, adding random slopes by subject for preceding trial, current trial, and their interaction, and using REML for linear mixed-effects models of SOT, approximating *p* values with Satterthwaite’s degrees of freedom method, as recommended by Luke (2017). The model of accuracy failed to converge with the full random effects structure, so random effects were simplified as described above.

### Data availability

Stimuli, presentation scripts, data, and analysis code are available online (https://osf.io/un5bd/?view_only=5ad295a80563422ca7d706e486396c94).

## Results

### Results for congruency sequence effects

The hypothesis that conflict between the spoken word and picture engaged cognitive control to benefit subsequent word recognition in noise was tested by examining congruency sequence effects on word recognition accuracy and SOTs at + 6 and + 8 dB SNR.

#### Word recognition accuracy exhibits congruency sequence effects

**Fig. 2:**
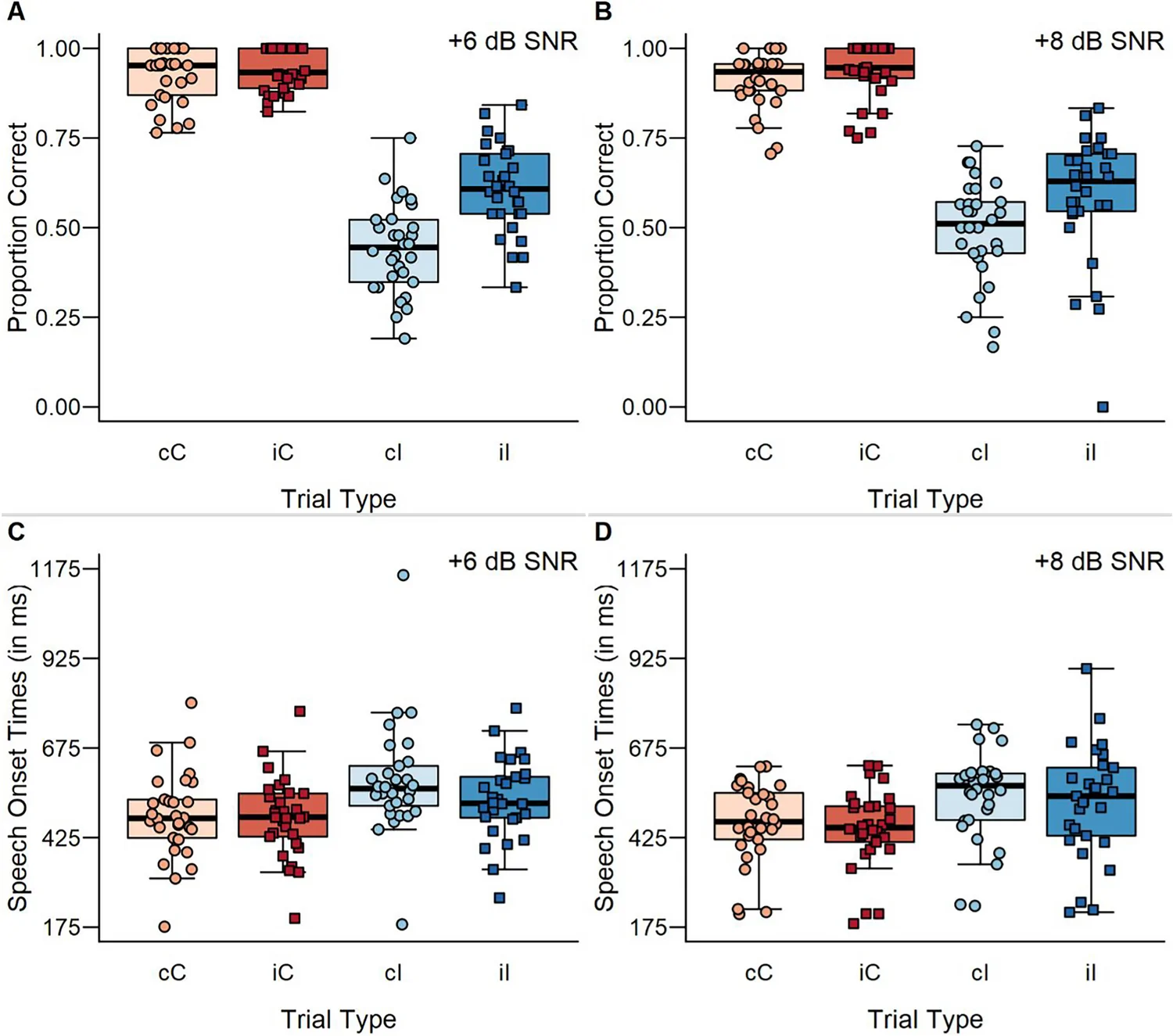
Word recognition accuracy and SOTs by trial type for + 6 **(A, C)** and + 8 dB SNR **(B, D).** *Note*. C = congruent, I = incongruent. Trial type is denoted by color and shape: cC (light red circles), iC (dark red squares), cI (light blue circles), and iI (dark blue squares), where the first letter represents preceding trial type and the second the current trial type

Model results are reported in Table 2, with accuracy for + 6 and + 8 dB SNR conditions shown in Fig. 2A–B. The model that converged was REC = PREC × CURR × SNR + (1 | SUB). Its fixed effects explained 32% of the variance in accuracy, with relatively little additional variance explained by the random subject intercept (total *R*^2^ = 0.33). Model results revealed that, when the preceding trial was congruent, accuracy was significantly lower for current incongruent than congruent trials (CURR: I estimate), showing that a mismatching picture reduced word recognition. Importantly, this interference effect was significantly smaller following a preceding incongruent than a preceding congruent trial (PREC: i × CURR: I estimate), supporting the hypothesis that cognitive control benefits speech recognition in noise.

**Table 2:** Mixed-effects logistic regression model parameters estimating speech recognition accuracy across + 6 and + 8 dB SNRs

Fixed effects	Estimate	95% CI	SE	z-value	p
(Intercept)	2.50	[2.28, 2.72]	0.11	22.86	< 0.001^***^
PREC: i	0.15	[−0.23, 0.48]	0.18	0.84	0.40
CURR: I	−2.65	[−2.86, −2.40]	0.12	−22.18	< 0.001^***^
SNR	−0.11	[−0.33, 0.12]	0.11	−0.97	0.33
PREC: i × CURR: I	0.44	[0.08, 0.85]	0.20	2.18	0.03^*^
PREC: i × SNR	0.10	[−0.23, 0.43]	0.18	0.55	0.59
CURR: I × SNR	0.21	[−0.03, 0.45]	0.12	1.77	0.08
PREC: i × CURR: I × SNR	−0.22	[−0.62, 0.18]	0.20	−1.08	0.28

^*^ *p* < 0.05, ^***^ *p* < 0.001. Estimates are given as log odds. The reported model is REC = PREC × CURR × SNR + (1|SUB); none of the models with random slopes converged. Trial type was dummy coded with congruent trials as the reference level and SNR was mean centered; the intercept represents cC trials at + 7 dB SNR. 95% CI = bootstrapped 95% Confidence Interval; SE = standard error; PREC: i = preceding incongruent trial; CURR: I = current incongruent trial. The variance for the by subjects random intercept was 0.05 with an adjusted intraclass correlation coefficient of 0.014

Follow-up model contrasts for the interaction between preceding and current trial type at each SNR showed that the reduction in interference magnitude following a preceding incongruent trial was significant at + 6 dB SNR (*OR* = 1.93, *SE* = 0.56; *z* = 2.26, *p* = 0.02) but not at + 8 dB SNR (*OR* = 1.25, *SE* = 0.35, *z* = 0.80, *p* = 0.42). Nevertheless, the estimated marginal means exhibited similar patterns across the two SNRs. Specifically, the probability of a correct response was significantly lower (*OR* = 0.49, *SE* = 0.06; *z* =  − 5.54, *p* < 0.001) for cI trials (*M* = 0.44, *SE* = 0.02, 95% CI [0.40, 0.48]) than for iI trials (*M* = 0.61, *SE* = 0.03, 95% CI [0.56, 0.66]) at + 6 dB SNR, and it was also significantly lower (*OR* = 0.62, *SE* = 0.08; *z* = −3.74, *p* = 0.001) for cI trials (*M* = 0.49, *SE* = 0.02, 95% CI [0.45, 0.53]) than for iI trials (*M* = 0.61, *SE* = 0.03, 95% CI [0.56, 0.66]) at + 8 dB SNR. The difference in the probability of a correct response (*OR* = 0.95, *SE* = 0.25; *z* =  − 0.20, *p* = 0.997) between cC trials (*M* = 0.93, *SE* = 0.01, 95% CI [0.91, 0.95]) and iC trials (*M* = 0.94, *SE* = 0.01, 95% CI [0.90, 0.96]) was not significant at + 6 dB SNR, nor did cC (*M* = 0.92, *SE* = 0.01, 95% CI [0.89, 0.94]) and iC trials (*M* = 0.93, *SE* = 0.01, 95% CI [0.90, 0.96]) differ at + 8 dB SNR (*OR* = 0.78, *SE* = 0.19; *z* =  − 1.00, *p* = 0.75). Thus, results show a boost in accuracy following conflict on incongruent but not congruent trials that occurred across + 6 and + 8 dB SNRs. The same pattern of results was observed when including posterror trials in the analyses, except that the interaction between preceding and current trial type at + 8 dB SNR reached significance (see Supplementary Tables 1 and 2).

Taken together, results demonstrate significant postconflict improvements in word recognition accuracy that emerged selectively on incongruent trials for both SNRs. This pattern is consistent with the classic congruency sequence effect, suggesting adaptive improvements in resolving conflict between a picture and a spoken word following incongruent trials during speech recognition in noise. The magnitude of the postconflict improvement, measured by iI – cI trial accuracy, appeared to be somewhat larger for the + 6 dB SNR than the + 8 dB SNR (17%-points versus 11%-points, respectively), as the interaction for preceding and current trial type only reached significance for the lower SNR condition.

#### SOTs exhibit congruency sequence effects

Model results are reported in Table 3, with SOTs at + 6 and + 8 dB SNR shown in Fig. 2C–D. The model that converged was SOT = PREC × CURR × SNR + (1 + PREC + CURR | SUB). Its fixed effects explained only 4% of the variance in SOTs, with considerably more variance explained by random subject effects (total *R*^2^ = 0.40). Model results showed that, following a congruent trial, SOTs were significantly slower for current incongruent than current congruent trials (CURR: I estimate). As with the accuracy results, this interference effect was significantly smaller following a preceding incongruent than a preceding congruent trial (PREC: i × CURR: I estimate).

**Table 3:** Mixed-effects linear regression model parameters estimating SOTs across + 6 and + 8 dB SNRs

Fixed effects	Estimate	95% CI	SE	t	df	p
(Intercept)	476.84	[445.20, 506.91]	14.58	32.71	58.74	< 0.001^***^
PREC: i	−9.91	[−24.12, 4.51]	7.29	−1.36	148.34	0.18
CURR: I	80.82	[60.38, 98.33]	10.20	7.92	83.42	< 0.001^***^
SNR	−10.71	[−39.42, 20.79]	14.58	−0.74	58.73	0.47
PREC: i × CURR: I	−22.14	[−43.76, −1.23]	11.28	−1.96	2884.71	0.049^*^
PREC: I × SNR	−4.42	[−19.10, 9.42]	7.29	−0.61	148.60	0.55
CURR: I × SNR	−10.66	[−32.86, 9.29]	10.20	−1.05	83.54	0.30
PREC: i × CURR: I × SNR	19.29	[−3.98, 41.31]	11.29	1.71	2884.55	0.09

^*^ *p* < 0.05, ^***^ *p* < 0.001. Estimates are given in ms. Trials with word recognition errors and those following errors are excluded from analyses. Trial type was dummy coded with congruent trials as the reference level, and SNR was mean centered; the intercept represents cC trials at + 7 dB SNR. 95% CI = bootstrapped 95% Confidence Interval; SE = standard error; PREC: i = preceding incongruent trial; CURR: I = current incongruent trial. The variances for the by subjects random effects were 11,614.9 for the intercept, 180 for PREC: i slope, and 2962.5 for the CURR: I slope with an adjusted intraclass correlation coefficient of 0.374

Examining model contrasts at each SNR revealed that the interaction between preceding and current trial type was significant at + 6 dB SNR (B =  − 42, *SE* = 16.2); *t*(2879) =  − 2.58, *p* = 0.01, but not at + 8 dB SNR (B =  − 3, *SE* = 15.7); *t*(2888) =  − 0.20, *p* = 0.84. Pairwise-comparisons of estimated marginal means confirmed that postconflict improvements in SOTs occurred for + 6 but not + 8 dB SNR. At + 6 dB SNR, SOTs were significantly slower (B = 47, *SE* = 12.9); *t*(332.5) = 3.66, *p* = 0.002, in response to cI trials (*M* = 579, *SE* = 23.8, 95% CI [532, 627]) than iI trials (*M* = 532, *SE* = 24.2, 95% CI [484, 581]), demonstrating faster responding on incongruent trials that followed conflict. Such postconflict performance improvements were not observed for congruent trials, as the difference in SOTs between cC trials (*M* = 488, *SE* = 20.6, 95% CI [447, 529]) and iC trials (*M* = 482, *SE* = 21.2, 95% CI [440, 525]) was not significant (B = 5, *SE* = 10.4); *t*(155.8) = 0.52, *p* = 0.95).

A different pattern emerged at + 8 dB SNR. Here, SOTs in response to cI trials (*M* = 537, *SE* = 23.6, 95% CI [489, 584]) and iI trials (*M* = 519, *SE* = 24.3, 95% CI [471, 568]) were not significantly different (B = 17, *SE* = 12.5); *t*(271.0) = 1.40, *p* = 0.50, showing little evidence of faster responses following conflict at + 8 dB SNR. Faster postconflict responses were also not observed for congruent trials, as there was no significant difference (*M* = 14, *SE* = 10.2); *t*(141.7) = 1.39, *p* = 0.51, in SOTs in response to cC trials (*M* = 466, *SE* = 20.6, 95% CI [425, 508]) versus iC trials (*M* = 452, *SE* = 21.1, 95% CI [410, 494]).

The same pattern of results was observed for each SNR when including posterror trials in analyses (see Supplementary Tables 3 and 4), demonstrating that removing trials did not substantially change the results.

In summary, participants were slower for incongruent than congruent trials across SNRs. They also became significantly faster at recognizing words on incongruent trials following conflict. However, follow-up comparisons demonstrate that the congruency sequence effect in SOTs only emerged at + 6 dB SNR. While there was a significant improvement of 47 ms from cI to iI trials at + 6 dB SNR, this effect dropped to 17 ms and was nonsignificant at + 8 dB SNR.

So far, we have observed congruency sequence effects in both accuracy and SOTs during word recognition in noise in a new dataset including higher SNRs than previously tested (Teubner-Rhodes et al., 2024). Sequential performance adjustments were driven by better performance on iI than cI trials, supporting the notion that preceding conflict engages cognitive control resources to improve speech recognition in noise. These effects appeared to be stronger at + 6 dB SNR than at + 8 dB SNR. We explicitly test the extent to which SNR modulates congruency sequence effects in the next section.

### Results for SNR effects

The hypothesis that congruency sequence effects on word recognition accuracy and SOTs are either reduced or follow an inverted-U pattern with increasing SNR was tested by comparing models with and without linear and quadratic SNR effects and their interactions with preceding and current trial type across + 4, + 6, and + 8 dB SNR levels (+ 4 dB SNR data are from Teubner-Rhodes et al., 2024). A three-way interaction between SNR and preceding and current trial type would show that congruency sequence effects are modulated by SNR, supporting the idea that the degree to which cognitive control benefits speech recognition in noise depends on intelligibility levels.

#### SNR modulates interference effects in word recognition accuracy

Results of the model comparison indicated that the model containing the linear SNR term and its interactions with preceding and current trial type significantly improved model fit (AIC = 5970.5); χ^2^(4) = 19.27, *p* < 0.001, over the base model without an SNR term (AIC = 5981.8). The model containing the quadratic SNR term did not further improve model fit (AIC = 5977.9); χ^2^(4) = 0.60, *p* = 0.96.

**Fig. 3:**
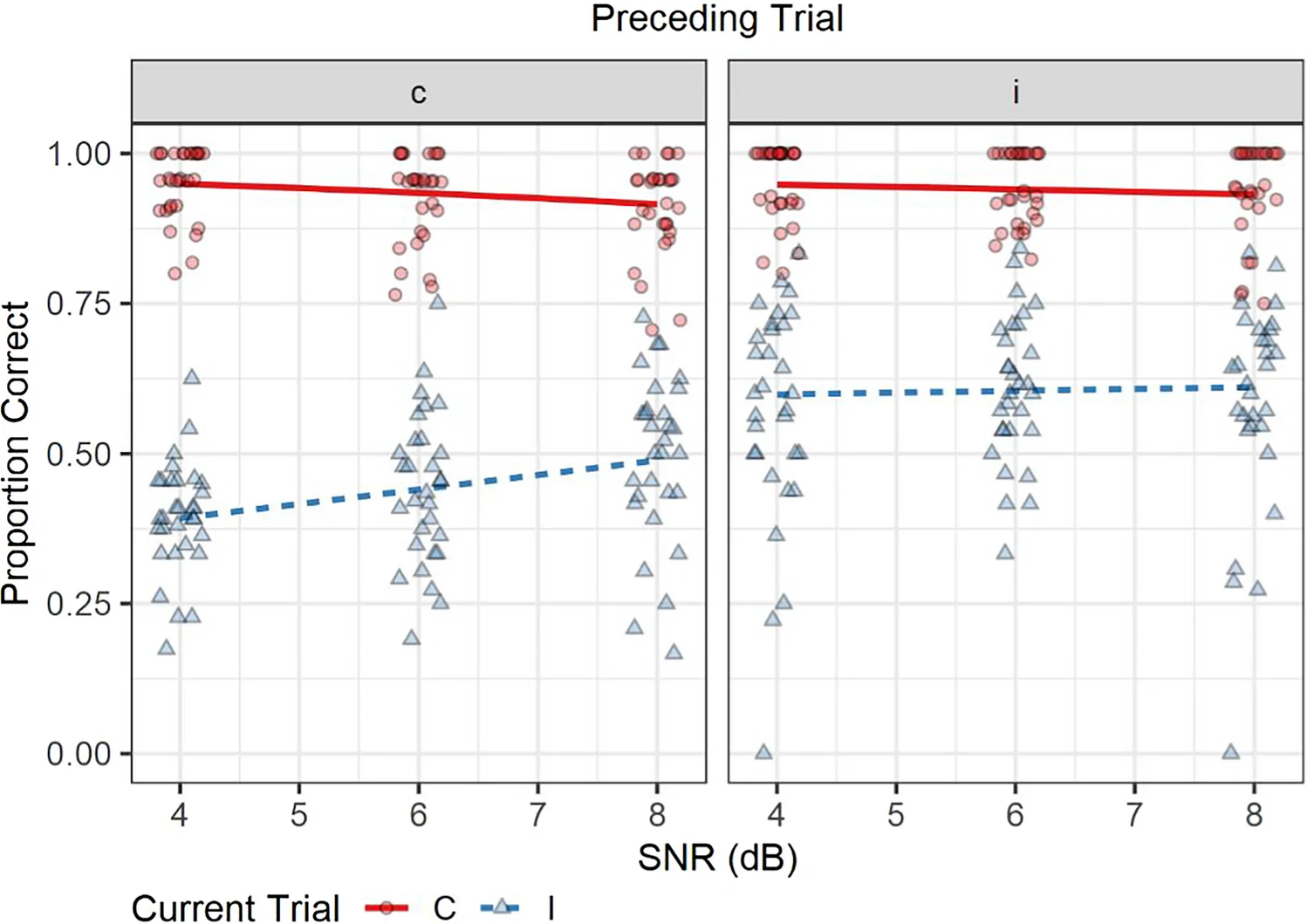
SNR effects on word recognition accuracy by preceding and current trial type. *Note*. C = congruent; I = incongruent. Lines represent model predictions for proportion correct word recognition for cC (left panel, solid red line), cI (left panel, dotted blue line), iC (right panel, solid red line), and iI trials (right panel, dotted blue line), where the first letter refers to preceding trial type and the second to current trial type. Points represent observed mean proportion correct for individual participants for cC (left panel, red circles), cI (left panel, blue triangles), iC (right panel, red circles), and iI trials (right panel, blue triangles). Data points were jittered along the x-axis for visualization

The results of the best-fitting model are presented in Table 4, and the fitted estimates for proportion correct word recognition are plotted in Fig. 3. The combined fixed effects of preceding trial, current trial, SNR, and their interactions explained 36% of the variance in word recognition accuracy, with little additional variance explained by the random intercept for subjects (total *R*^2^ = 0.37). As observed previously, word recognition accuracy was significantly worse for incongruent than congruent trials when the preceding trial was congruent (CURR: I estimate), but this effect was smaller when the preceding trial was incongruent (PREC: i × CURR: I estimate), demonstrating significant congruency sequence effects. Importantly, a significant interaction between current trial type and SNR (CURR: I × SNR estimate) showed that increasing the SNR decreased the interference effect when the preceding trial was congruent (i.e., the difference between word recognition accuracy on cC and cI trials).

**Table 4:** Parameters of best-fitting mixed-effects logistic regression model estimating SNR effects on speech recognition accuracy

Fixed effects	Est	95% CI	SE	z-value	p
(Intercept)	2.66	[2.46, 2.86]	0.09	28.23	< 0.001^***^
PREC: i	0.10	[−0.20, 0.41]	0.16	0.64	0.52
CURR: I	−2.90	[−3.09, −2.68]	0.10	−28.41	< 0.001^***^
SNR	−0.14	[−0.25, −0.01]	0.06	−2.41	0.02^*^
PREC: i × CURR: I	0.57	[0.23, 0.92]	0.17	3.29	< 0.001^***^
PREC: i × SNR	0.06	[−0.14, 0.24]	0.09	0.68	0.50
CURR: I × SNR	0.24	[0.11, 0.37]	0.06	3.81	< 0.001^***^
PREC: i × CURR: I × SNR	−0.15	[−0.35, 0.07]	0.10	−1.43	0.15

^*^ *p* < 0.05, ^***^ *p* < 0.001. Estimates are given as log odds. The best-fitting model was REC = PREC × CURR × SNR + (1|SUB); none of the models with random slopes converged. Trial type was dummy coded with congruent trials as the reference level and SNR was mean centered; the intercept represents cC trials at + 6 dB SNR. Est. = estimate; 95% CI = bootstrapped 95% Confidence Interval; SE = standard error; PREC: i = preceding incongruent trial; CURR: I = current incongruent trial. The variance of the by subjects random intercept was 0.04 with an adjusted intraclass correlation coefficient of 0.013

Follow-up contrasts of estimated marginal means showed that the probability of recognizing a cI trial at + 4 dB SNR (*M* = 0.39, *SE* = 0.02, 95% CI [0.36, 0.43]) was significantly lower (*OR* = 0.68, *SE* = 0.08; *z* =  − 3.21, *p* = 0.007) than the probability of recognizing a cI trial at + 8 dB SNR (*M* = 0.49, *SE* = 0.02, 95% CI [0.45, 0.53]), demonstrating a + 2.5%-point improvement in word recognition per 1 dB increase in SNR for cI trials. The probability of recognizing cC trials with increasing SNR trended in the opposite direction, with a numerically higher probability (OR = 1.73, *SE* = 0.40; *z* = 2.41, *p* = 0.08) of recognizing a cC trial at + 4 dB SNR (*M* = 0.95, *SE* = 0.01, 95% CI [0.93, 0.96]) than at + 8 dB SNR (*M* = 0.92, *SE* = 0.01; 95% CI [0.89, 0.93]), although this effect did not reach significance. In contrast, SNR did not significantly affect word recognition for either congruent or incongruent trials when the preceding trial was incongruent. Specifically, the probability of recognizing an iI trial at + 4 dB SNR (*M* = 0.60, *SE* = 0.02, 95% CI [0.55, 0.64]) was not significantly different (*OR* = 0.95, *SE* = 0.15; *z* =  − 0.34, *p* = 0.99) than the probability of recognizing an iI trial at + 8 dB SNR (*M* = 0.61, *SE* = 0.02, 95% CI [0.56, 0.66]). Additionally, the probability of recognizing an iC trial did not significantly change (*OR* = 1.34, *SE* = 0.42, *z* = 0.94, *p* = 0.79) from + 4 dB SNR (*M* = 0.95, *SE* = 0.01, 95% CI [0.92, 0.97]) to + 8 dB SNR (*M* = 0.93, *SE* = 0.01, 95% CI [0.90, 0.95]). Thus, the reduced interference effect with increasing SNR was primarily driven by an increase in word recognition accuracy for cI trials with higher SNRs, although there was also a slight decline in accuracy for cC trials. Participants were less affected by picture congruency as listening conditions became easier, but only when the preceding trial was congruent.

Although this pattern is consistent with a reduction in congruency sequence effects with increasing SNR, the three-way interaction between preceding trial type, current trial type, and SNR did not reach significance. This suggests that any effect of SNR on the magnitude of postconflict performance adjustments is modest. The same pattern of results was observed when including trials following errors in analyses (see Supplementary Table 5).

#### SOTs are largely unaffected by SNR

Results of the model comparison indicated that the base model (AIC = 58,600), which contained effects of preceding and current trial type but not SNR, was the best-fitting model. Adding the linear (AIC = 58,601), χ^2^(4) = 7.25, *p* = 0.12, or quadratic SNR terms (AIC = 58,605), χ^2^(4) = 3.71, *p* = 0.45, and their interactions did not significantly improve model fit.

Parameters of the best-fitting model are reported in Table 5. The combined fixed effects of preceding and current trial type explained 4% of the variance in SOTs, with an additional 36% explained by the random intercept by subjects. There was a significant positive effect of current trial due to slower SOTs for incongruent than congruent trials when the preceding trial was congruent (CURR: I effect). Additionally, there was a significant interaction between preceding and current trial (PREC: i × CURR: I estimate). Follow-up contrasts of estimated marginal means found that participants were significantly slower (B = 29, *SE* = 8.2), *t*(85.5) = 3.48, *p* = 0.004, on cI trials (*M* = 565, *SE* = 13.9, 95% CI [537, 592]) than on iI trials (*M* = 536, *SE* = 13.8, 95% CI [509, 564]), demonstrating a postconflict improvement in SOTs. In contrast, there was no significant difference in SOTs (B = 6, *SE* = 5.9), *t*(556.6) = 1.11, *p* = 0.69, between cC trials (*M* = 476, *SE* = 11.7, 95% CI [453, 499]) and iC trials (*M* = 469, *SE* = 11.5, 95% CI [446, 492]). Thus, participants experienced less interference from an incongruent picture during speech recognition in noise following conflict, making faster postconflict responses selectively on incongruent trials.

**Table 5:** Parameters of best-fitting mixed-effects linear regression model estimating SNR effects on SOTs

Fixed effects	Estimate	95% CI	SE	t-value	df	p
(Intercept)	475.89	[451.48, 499.28]	11.70	40.66	89.36	< 0.001^***^
PREC: i	−6.48	[−16.80, 4.87]	5.87	−1.11	556.59	0.27
CURR: I	88.90	[67.52, 108.36]	10.42	8.54	86.97	< 0.001^***^
PREC: i × CURR: I	−22.02	[−40.67, −2.06]	10.24	−2.15	137.06	0.03^*^

^*^ *p* < 0.05, ^***^ *p* < 0.001. Estimates are given in ms. The best-fitting model was REC = PREC × CURR + (1 + PREC × CURR |SUB), which includes the full random effects structure. Trials with word recognition errors and those following errors are excluded from analyses. Trial type was dummy coded with congruent trials as the reference level; the intercept represents cC trials. 95% CI = bootstrapped 95% Confidence Interval; SE = standard error; PREC: i = preceding incongruent trial; CURR: I = current incongruent trial. The variance was 11,382 for the by-subjects intercept and 177, 6525, and 1774 for random slopes of preceding trial, current trial, and their interaction, respectively, with an adjusted intraclass correlation coefficient of 0.368

The effect of SNR was not included in best-fitting model of SOTs, as it did not significantly improve model fit. This suggests that SNR did not affect how quickly participants repeated the spoken word. When including trials that followed errors in analyses, the model that contained the linear SNR term and its interactions was a marginally better fit (AIC = 81,716); χ^2^(4) = 9.16, *p* = 0.057, than the base model without SNR (AIC = 81,717). Nevertheless, none of the SNR effects in this model approached significance (see Supplementary Table 6). Overall, results demonstrate congruency sequence effects in SOTs that appear to remain relatively consistent across SNRs from + 4 to + 8 dB.

#### Retrospective power analysis for interaction of preceding trial, current trial, and SNR

As the present study is the first to investigate how SNR modulates congruency sequence effects, no a priori estimate was available for the effect size of the interaction between preceding trial, current trial, and SNR. The results suggest that our study may have been underpowered to detect this interaction for our primary outcome measure of interest, word recognition accuracy. At the suggestion of reviewers, we conducted a retrospective power analysis using the *simr* package to test combinations of SNRs and sample sizes that would provide at least 80% power to detect a significant interaction between preceding trial, current trial, and SNR for word recognition accuracy. We applied the effect size of − 0.15 obtained in the present study to the full experimental design containing 24 trials per condition using the model that converged in our analysis of SNR effects, REC = PREC × CURR × SNR + (1 | SUB). Using the intraclass correlation coefficient estimated from the original data (ICC = 0.026), we used the extend function to simulate SNR values from + 2 to 14 dB in 2, 3 or 4 dB increments and the “powerCurve” function to systematically test power for different numbers and ranges of SNR conditions. We also used “powerCurve” to test the effect of increasing the number of participants per SNR condition from 30 to 60 in increments of 6. Our initial testing of different designs set the number of simulations to *n* = 50 for increased speed. After selecting a design with sufficient power (see below), we conducted 500 simulations to obtain a more precise power estimate.

This analysis allowed us to identify the design that required the fewest additional observations to achieve at least 80% power for detecting a significant three-way interaction at α = 0.05 for the effect size observed in the present study. Testing + 2, 6, and 10 dB SNRs with samples of *n* = 31, 30, and 30, respectively, would provide 95% power (95% CI [92%, 96%]) to detect a three-way interaction of − 0.15 without adding any observations—this design increases the SNR range without increasing sample size or number of SNR conditions. Increasing the SNR step-size to + 4 dB SNR may be an efficient approach for achieving the necessary power to detect significant moderations of SNR on congruency sequence effects in word recognition accuracy.

## Discussion

We manipulated conflict during speech recognition in noise at + 6 and + 8 dB SNRs by presenting pictures that matched or were phonological neighbors of the current target word. The conflict manipulation resulted in lower word recognition accuracy and slower response times at both SNRs. Importantly, we replicated the postconflict benefit in word recognition accuracy observed at a lower SNR by Teubner-Rhodes et al. (2024). Findings also extended postconflict improvements in speech recognition to response times for the first time. Finally, we found that participants experienced less interference, measured as the difference in word recognition accuracy between congruent and incongruent trials, as SNR increased from + 4 to + 8 dB. However, SNR did not significantly modulate congruency sequence effects during speech recognition in noise. Theoretical implications of these postconflict improvements and SNR effects on interference during speech recognition in noise are discussed below.

### Conflict effect and postconflict improvements support ELU and conflict monitoring

Our results demonstrate how visual referents can influence the recognition of spoken words. Specifically, participants were slower and less accurate at recognizing words in background noise when viewing an incongruent picture of a phonological neighbor compared with a congruent picture. This effect can be explained by the ELU’s RAMPHO process (Rönnberg et al., 2013, 2019, 2021), in which the picture of the phonological neighbor would be immediately bound to the spoken word to create a multimodal phonological representation. For congruent trials, the additional phonological evidence provided by the picture should create a clear representation that is a close match with the lexical target, passing the threshold for correct recognition—this is evidenced by the consistently high (> 90%) recognition for congruent words. However, incongruent trials create an ambiguous representation that partially matches with the lexical target, the pictured phonological neighbor, and other neighbors that sound similar to the spoken input. Such ambiguous representations make the spoken word more difficult to recognize, slowing responses and reducing recognition accuracy.

Improvements in recognizing speech in noise following conflict provide support for conflict monitoring theory (Botvinick et al., 2001). Participants were better at resolving conflict between the spoken word and a picture of its phonological neighbor after another incongruent trial. That participants became faster in addition to becoming more accurate at recognizing words following conflict indicates that results cannot be due to a speed–accuracy trade-off: rather than slowing down their responses to ensure correct word recognition following conflict, participants sped up and were still better able to understand words in noise. This suggests that conflict engaged cognitive control to allow fast and accurate selection of the target among competing lexical representations. These postconflict performance improvements in speech recognition join a large body of work showing congruency sequence effects in classic cognitive control tasks such as Simon, Stroop, and flanker (e.g., Akçay & Hazeltine, 2011; Egner & Hirsch, 2005; Gratton et al., 1992; Jiménez & Méndez, 2013; Notebaert & Verguts, 2008). While these studies show postconflict performance adjustments that support conflict monitoring theory, researchers have pointed out that these tasks are often confounded by feature repetitions and/or stimulus–response contingencies that may underlie congruency sequence effects instead of conflict (Schmidt, 2013, 2019). Our findings show that congruency sequence effects can occur in the absence of such confounds and that conflict monitoring applies to speech recognition in noise, at least at intermediate difficulty levels where speech recognition is challenging but possible.

Most prior studies have examined correlations between performance on cognitive control tasks and tests of speech recognition to evaluate the role of cognitive control in understanding speech in noisy environments (e.g., Sommers & Danielson, 1999; Taler et al., 2010). While this work is important, it cannot show that cognitive control plays a causal and necessary role in recognizing speech in noise. A few studies have conducted cognitive training to assess whether enhancing cognitive abilities improves speech recognition in noise, but their results have been mixed. Cogmed working memory training (Klingberg et al., 2002, 2005) did not benefit speech recognition in noise in any of a variety of stimuli and background noise conditions in older adults with a range of hearing levels (Henshaw et al., 2022; Wayne et al., 2016). However, the training tasks in these studies primarily tapped storage components of working memory and showed little transfer even to other working memory tasks, so they may not have improved cognitive control abilities per se. Training paradigms that combined auditory discrimination and cognitive tasks have successfully improved performance on speech recognition in noise tasks, auditory working memory, and inhibitory control (Anderson et al., 2013; Sweetow & Sabes, 2006), but it is unclear the extent to which these training benefits can be attributed to the cognitive versus auditory aspects of training. We show that inducing cognitive control via conflict *within an individual* improves subsequent speech recognition in noise. As each individual serves as their own control, and cognitive control is manipulated across trials, results cannot be attributed to confounding variables that might affect both cognitive control and speech recognition abilities.

Postconflict improvements in recognizing speech in noise are also broadly consistent with the ELU (Rönnberg et al., 2013, 2019, 2021). The ELU holds that working memory, including an executive component for directing attention and inhibiting irrelevant information, plays an important role in understanding speech whenever the phonological representation supported by the speech input is not a good match for the lexical representation in long-term memory. In our experimental paradigm, the picture, which is presented 500 ms prior to the spoken word, yields a prediction about the identity of the upcoming target that is hypothesized to prime RAMBPHO to restrict the space of phonological representations (Rönnberg et al., 2019, 2021). This prediction is ultimately correct for congruent trials and incorrect for incongruent trials. The mismatch created by the misleading prediction from the visual stream and noise in the auditory input requires cognitive control to select the closest match for the speech signal and suppress competitors—we argue that cognitive control rather than working memory is relevant here because isolated word recognition does not require maintaining representations for ongoing interpretation of a sentence. Conflict monitoring theory (Botvinick et al., 2001) includes postdiction processes, where cognitive control adjusts task weights to increase focus on task-relevant information. In the present study, this may involve tuning attention to the speech signal over background noise, reducing attention to the picture, or increasing activation of the target word in the mental lexicon relative to phonological competitors.

### SNR effects reveal cognitive demands of low speech intelligibility

Higher SNRs significantly reduced the magnitude of interference from the picture during speech recognition in noise. Following a congruent trial, higher SNRs increased word-recognition accuracy for incongruent trials and slightly decreased it for congruent trials. This finding is consistent with prior work showing that the word recognition advantage from semantically congruent text cues relative to incongruent and nonsense cues is larger at lower SNRs (Zekveld et al., 2011). Increasing the SNR may have reduced listening demands, making cognitive-control resources more available and enabling better phonological conflict resolution overall. Higher SNRs may have also decreased reliance on visual information to help decode the noisy auditory signal, especially if audiovisual integration is cognitively taxing in listening environments where the visual information is not necessary for performing the task (Mishra et al., 2013). Although the auditory input was always the task-relevant modality in the present study, the pictures provide information that supports correct recognition on congruent trials, especially at lower SNRs when intelligibility is poor. As SNR increases, the speech input becomes clearer, so the listener may shift their focus to the task-relevant auditory modality. As the picture exerts less influence on spoken word recognition, performance on incongruent trials would improve while performance on congruent trials would worsen. Prior work has found that interference effects tend to be small when the irrelevant and relevant stimulus dimensions have similar discriminability and increase as the irrelevant dimension becomes more discriminable than the target dimension (see Algom et al., 2021, for discussion). Consistent with the present study, interference from the task-irrelevant modality is largest when discriminability of the target modality is low.

Larger interference effects at poorer intelligibility levels suggests the role of cognitive control in recognizing speech may increase at lower SNRs. Cognitive control may be less available to resolve conflict between a spoken word and a phonologically related picture as listening demands increase. Nevertheless, SNR did not modulate congruency sequence effects, suggesting that engaging cognitive control offers similar word recognition benefits from + 4 to + 8 dB SNR. This could indicate that the tested range of SNRs produce intermediate intelligibility levels where cognitive control is likely to have its maximal benefit (Rönnberg et al., 2013). Indeed, our exploratory power analysis suggested that extending the SNR range from + 2 to + 10 dB SNR may provide sufficient power to detect significant moderation of SNR on congruency sequence effects.

Our results join a growing body of evidence supporting a role for cognitive control in understanding speech in background noise. A meta-analysis by Dryden et al. (2017) investigating the relationship between different cognitive abilities and recognition of speech in noise found that, across studies of adults with normal hearing to moderate hearing loss, the association between inhibitory control and speech recognition in noise was *r* = 0.34. Associations between cognitive abilities and speech recognition in noise generally increased as the amount of informational masking in the background noise increased; however, there was insufficient data to demonstrate this effect of masker type for inhibitory control specifically. As the 12-talker babble used in the present study primarily induces energetic masking, this suggests that cognitive control effects on speech recognition in noise may be even larger for fewer competing talkers that elicit greater informational masking.

An association between cognitive control and speech recognition in noise may be exacerbated in older adults or in individuals with uncorrected hearing loss. In a study of Cantonese-speaking older adults with hearing loss, lower executive function was associated with higher (i.e., worse) thresholds for speech recognition in noise in a group that had never used hearing aids but not in a group of hearing aid users (Chen et al., 2022). Van Knijff et al. (2018) found that better inhibitory control, as indicated by smaller Simon effects, was associated with a higher likelihood of recognizing phonemes in noise for both words and sentences in older adults with hearing loss but not in younger adults or normal hearing older adults. The ELU also predicts poorer performance for those with hearing loss (Rönnberg et al., 2013, 2019, 2021): Relative to listeners with normal hearing, those with hearing loss will experience more frequent and more severe mismatches between the input and the lexicon requiring executive control to enable correct recognition. It is remarkable, then, that we observed that inducing cognitive control improved speech recognition in noise in a sample of healthy young adults with normal hearing, as the importance of cognitive control for successful speech recognition may be reduced in this population. Our findings join prior work that has observed a relationship between inhibitory control abilities and speech recognition in noise even in normal hearing young adults (Stenbäck et al., 2015).

### Limitations and future directions

We acknowledge that our results only apply to the range of SNRs tested. Although the need for cognitive control was not significantly affected by SNRs from + 4 to + 8 dB, it is still possible that its role may decrease at more—or less—favorable SNRs. However, we caution that SNRs that are much further outside of the range employed here may be subject to floor or ceiling effects. The postconflict boost can be as large as + 17%-points, which means that scoring higher than 83% on cI trials could limit participants’ opportunity to improve their recognition following conflict—our top-performing participants approached but did not reach this boundary, with two participants scoring 75% and 73% on cI trials in the + 6 and + 8 dB conditions, respectively. As recognition improves, effects may shift from recognition accuracy to speech onset times, with heightened control enabling faster rather than more accurate recognition. Future studies could examine a larger range of SNRs to evaluate how the role of cognitive control changes as speech becomes more intelligible or less intelligible.

The present study did not manipulate the type of background masker, as the focus was on the influence of SNR rather than masker types on cognitive control. It is possible that some masker types may engage cognitive control to a different extent or in a different way than others. The multitalker babble used in the present study provides an ecologically valid simulation of real-world listening conditions. Because the babble contains 12 talkers, it also prevents listeners from understanding particular words in the background noise that could be confused with the speech signal. This is useful for interpretation of the present study because it suggests that phonological competition arises from uncertainty in the perception of phonemes in the speech signal rather than confusability with words in the noise signal. A noise masker consisting of one or two competing speakers may require cognitive control to a greater extent (Dryden et al., 2017), due to the additional competition that can arise from words in the noise signal. On the other hand, fewer competing talkers allows for more glimpses in which the speech signal is less affected by the masker and therefore more intelligible (Cooke, 2006), which may reduce the need for cognitive control. Comparing cognitive control demands for different types of background noise is an important area for future research.

It remains unclear how cognitive control operates to benefit speech recognition in noise. In the present task, cognitive control may focus attention on the relevant speech signal, decrease attention to the irrelevant picture, and/or resolve conflict between the target word and competitors in the mental lexicon. Although we provided preliminary evidence that cognitive control offers a similar benefit across SNRs, potentially favoring a role in conflict resolution over attention to the speech signal, we may also have been underpowered to detect changes in postconflict performance across + 4 to + 8 dB SNR. Prior work provides evidence that cognitive control does not primarily operate by reducing attention to the picture in the SPRiNT paradigm: following conflict, word recognition errors were less likely to be related to the picture and more likely to be a different neighbor of the spoken word, while errors that were not phonologically related to the word or picture remained unchanged (Teubner-Rhodes et al., 2024). This suggests that cognitive control may operate by focusing attention on the speech signal or by resolving phonological conflict; however, additional research is necessary to fully understand the mechanisms by which cognitive control improves speech recognition in noise in the presence of visual referents.

## Conclusions

We observed that speech recognition in noise improved following phonological conflict induced by a picture of a neighbor of the target word. This was true even when omitting trials that followed errors, indicating that this is a postconflict rather than a posterror effect. Higher SNRs improved speech recognition for incongruent trials, reducing the amount of interference from a phonologically related picture. However, better intelligibility did not significantly affect the size of the postconflict boost. Our results demonstrate a conflict monitoring effect during speech recognition in noise that is somewhat robust to speech intelligibility. These findings suggest that triggering cognitive control may directly benefit recognition of speech in noisy environments, perhaps by improving selection of the target word among activated phonological competitors in the mental lexicon.

## Supplementary Information

Below is the link to the electronic supplementary material.

Supplementary file1 (DOCX 373 kb)

## Data Availability

Stimuli, presentation scripts, and data are available online (https://osf.io/un5bd/?view_only=5ad295a80563422ca7d706e486396c94). The study was not preregistered.

## Code availability

Analysis code is available online (https://osf.io/un5bd/?view_only=5ad295a80563422ca7d706e486396c94).
