# Supplementary material for: The effects of intelligibility on conflict resolution and monitoring during speech recognition in noise
Source: Atten Percept Psychophys. 2026 May 6;88(5):126. doi: 10.3758/s13414-026-03271-2 (PMC13149574; doi:10.3758/s13414-026-03271-2)
Supplement: Supplementary file 1 — Supplementary file1 (DOCX 373 kb) [file 13414_2026_3271_MOESM1_ESM.docx]

**Supplementary Table 1**

*Mixed-Effects Logistic Regression Model Parameters Estimating Speech Recognition Accuracy across +6 and +8 dB SNRs when Including Post-Error Trials*

| **Fixed effects** | **Estimate** | **95% CI** | **SE** | **z-value** | **p** |
| --- | --- | --- | --- | --- | --- |
| (Intercept) | 2.42 | [2.22, 2.60] | 0.10 | 23.70 | <0.001^***^ |
| PREC: i | 0.09 | [-0.19, 0.38] | 0.14 | 0.62 | 0.53 |
| CURR: I | -2.57 | [-2.75, -2.34] | 0.11 | -23.36 | <0.001^***^ |
| SNR | -0.08 | [-0.26, 0.12] | 0.10 | -0.75 | 0.46 |
| PREC: i × CURR: I | 0.58 | [0.25, 0.90] | 0.16 | 3.67 | <0.001^***^ |
| PREC: I × SNR | -0.02 | [-0.30, 0.24] | 0.14 | -0.11 | 0.91 |
| CURR: I × SNR | 0.18 | [-0.04, 0.38] | 0.11 | 1.63 | 0.10 |
| PREC: i × CURR: I × SNR | -0.08 | [-0.39, 0.26] | 0.16 | -0.50 | 0.62 |

*Note*. ^**^ p < 0.01, ^***^ p < 0.001. Estimates are given as log odds. The final model that converged was REC = PREC × CURR × SNR + (1|SUB). Trial type was dummy coded with congruent trials as the reference level and SNR was mean centered; the intercept represents cC trials at +7 dB SNR. The variance for the by-subjects random intercept was 0.08 with an adjusted intraclass correlation coefficient of 0.023. PREC: i = preceding incongruent trial; CURR: I = current incongruent trial. Results are comparable to those in the main text when excluding post-error trials, except that the interaction contrast of preceding and current trial reached significance for +8 dB SNR (odds ratio = 1.65, *SE* = 0.36; *z* = 2.31, *p* = 0.02) in addition to +6 dB SNR (odds ratio = 1.93, *SE* = 0.44; *z* = 2.87, *p* = 0.004).

**Supplementary Table 2**

*Estimated Marginal Means for Probability of Correct Word Recognition by Critical Trial Type when Including Post-Error Trials*

| **Cur.** | **Preceding Trial Type** | | | | | | **Pairwise**  **Contrasts** | | | |
| --- | --- | --- | --- | --- | --- | --- | --- | --- | --- | --- |
|  | **c** | | | **i** | | |  |  |  |  |
|  | *M* | *SE* | *95% CI* | *M* | *SE* | *95% CI* | *OR* | *SE* | *Z* | *p* |
| *+6 dB SNR* | | | | | | | | | | |
| C | 0.92 | 0.01 | [0.90, 0.94] | 0.93 | 0.01 | [0.91, 0.95] | 0.90 | 0.18 | -0.50 | 0.96 |
| I | 0.44 | 0.02 | [0.40, 0.48] | 0.63 | 0.02 | [0.58, 0.67] | 0.47 | 0.05 | -6.99 | <0.001^***^ |
| *+8 dB SNR* | | | | | | | | | | |
| C | 0.91 | 0.01 | [0.89, 0.93] | 0.92 | 0.01 | [0.89, 0.94] | 0.93 | 0.17 | -0.38 | 0.98 |
| I | 0.49 | 0.02 | [0.45, 0.53] | 0.63 | 0.02 | [0.59, 0.67] | 0.57 | 0.06 | -5.25 | <0.001^***^ |

*Note*. ^***^ < 0.001. Preceding c = preceding congruent; Preceding i = preceding incongruent; Cur. C = Current Congruent; Cur. I = Current Incongruent; OR = Odds Ratio.

**Supplementary Table 3**

*Mixed-effects linear regression model parameters estimating SOTs across +6 and +8 dB SNRs when Including Post-Error Trials*

| **Fixed effects** | **Estimate** | **95% CI** | **SE** | **t-value** | **df** | **p** |
| --- | --- | --- | --- | --- | --- | --- |
| (Intercept) | 477.5 | [450, 507] | 14.4 | 33.24 | 59.1 | <0.001^***^ |
| PREC: i | -8.9 | [-22, 2] | 6.2 | -1.43 | 122.8 | 0.15 |
| CURR: I | 85.4 | [63, 107] | 10.8 | 7.89 | 93.4 | <0.001^***^ |
| SNR | -11.6 | [-42, 14] | 14.4 | -0.80 | 59.1 | 0.42 |
| PREC: i × CURR: I | -27.8 | [-45, -8] | 9.5 | -2.94 | 4050.7 | 0.003^**^ |
| PREC: i × SNR | 3.1 | [-8, 15] | 6.2 | 0.51 | 122.8 | 0.61 |
| CURR: I × SNR | -10.5 | [-31, 11] | 10.8 | -0.97 | 93.4 | 0.34 |
| PREC: i × CURR: I × SNR | 10.7 | [-9, 29] | 9.5 | 1.13 | 4050.7 | 0.26 |

*Note*. ^**^ < 0.01, ^***^ < 0.001. Estimates are given in ms. Trials with word recognition errors are excluded from analyses. The model that converged was ONS = PREC + CURR + PREC × CURR + (PREC + CURR|SUB). Trial type was dummy coded with congruent trials as the reference level and SNR was mean centered; the intercept represents cC trials at +7 dB SNR. PREC: i = preceding incongruent trial; CURR: I = current incongruent trial. Variances for the by-subjects random effects were 11388 for the intercept, 327 for the preceding trial random slope, and 4049 for the current trial random slope with an adjusted intraclass correlation coefficient of 0.379.

**Supplementary Table 4**

*Estimated Marginal Means for SOTs by Critical Trial Type when Including Post-Error Trials*

| **Cur.** | **Preceding Trial Type** | | | | | | **Pairwise Contrasts** | | | | |
| --- | --- | --- | --- | --- | --- | --- | --- | --- | --- | --- | --- |
|  | **c** | | | **i** | | |  |  |  |  |  |
|  | *M* | *SE* | *95% CI* | *M* | *SE* | *95% CI* | *B* | *SE* | *df* | *t* | *p* |
| *+6 dB SNR* | | | | | | | | | | | |
| C | 489 | 20.3 | [448, 530] | 477 | 20.3 | [437, 518] | 12 | 8.7 | 121.6 | 1.38 | 0.52 |
| I | 585 | 24.0 | [537, 633] | 534 | 24.3 | [486, 583] | 51 | 15.4 | 319.6 | -6.22 | <0.001^***^ |
|  |  |  |  |  |  |  |  |  |  |  |  |
| *+8 dB SNR* | | | | | | | | | | | |
| C | 466 | 20.3 | [425, 502] | 460 | 20.3 | [420, 501] | 6 | 8.8 | 124.0 | 0.66 | 0.91 |
| I | 541 | 23.9 | [493, 589] | 518 | 24.4 | [469, 567] | 23 | 11.0 | 278.7 | 2.10 | 0.16 |

*Note*. ^***^ p < 0.001. Preceding c = preceding congruent; Preceding i = preceding incongruent; Cur. C = Current Congruent; Cur. I = Current Incongruent. Results are comparable to those in the main text when excluding post-error trials.

**Supplementary Table 5**

*Parameters of Best-Fitting Mixed-Effects Logistic Regression Model Estimating SNR Effects on Speech Recognition Accuracy when Including Post-Error Trials*

| **Fixed effects** | **Est.** | **95% CI** | **SE** | **z-value** | **p** |
| --- | --- | --- | --- | --- | --- |
| (Intercept) | 2.61 | [2.41, 2.77] | 0.09 | 28.87 | <0.001^***^ |
| PREC: i | 0.10 | [-0.16, 0.33] | 0.12 | 0.79 | 0.43 |
| CURR: I | -2.84 | [-3.02, -2.63] | 0.10 | -29.68 | <0.001^***^ |
| SNR | -0.15 | [-0.25, -0.03] | 0.05 | -2.70 | 0.007^**^ |
| PREC:i × CURR: I | 0.61 | [0.34, 0.92] | 0.14 | 4.46 | <0.001^***^ |
| PREC: i × SNR | -0.01 | [-0.16, 0.16] | 0.07 | -0.15 | 0.88 |
| CURR: I × SNR | 0.24 | [0.10, 0.35] | 0.06 | 4.08 | <0.001^***^ |
| PREC: i × CURR: I × SNR | -0.04 | [-0.22, 0.13] | 0.08 | -0.52 | 0.60 |

*Note*. ^**^ p < 0.01, ^***^ p < 0.001. Estimates are given as log odds. The reported model is REC = PREC × CURR × SNR + (1|ID); none of the models with random slopes converged. It explains 35% of the variance in word recognition accuracy, with 33% explained by fixed effects alone. Variance for the random intercept by subjects was 0.09 with an adjusted intraclass correlation coefficient of 0.026. Trial type was dummy coded with congruent trials as the reference level and SNR was mean centered; the intercept represents cC trials at +6 dB SNR. Est. = Estimate; PREC: i = preceding incongruent trial; CURR: I = current incongruent trial. The model with the linear SNR term and its interactions provided a significantly better fit (AIC = 8062.8; χ2(4) = 29.43, *p* < 0.001) than the one without the SNR term (AIC = 8084.2), while adding the quadratic SNR term did not further improve model fit (AIC = 8069.1; χ2(4) = 1.74, *p* = 0.78). The pattern of results matches those reported in the main text for the model that omitted post-error trials from analyses.

**Supplementary Table 6**

*Parameters of Best-Fitting Mixed-Effects Linear Regression Model Estimating SNR Effects on SOTs when Including Post-Error Trials*

| **Fixed effects** | **Estimate** | **95% CI** | **SE** | **t-value** | **df** | **p** |
| --- | --- | --- | --- | --- | --- | --- |
| (Intercept) | 475.6 | [453, 495] | 11.4 | 41.70 | 91.1 | <0.001^***^ |
| PREC: i | -5.7 | [-15, 3] | 4.9 | -1.17 | 188.5 | 0.24 |
| CURR: I | 91.6 | [73, 110] | 9.2 | 9.93 | 136.8 | <0.001^***^ |
| SNR | -1.4 | [-15, 12] | 7.0 | -0.20 | 90.9 | 0.84 |
| PREC × CURR: iI | -24.4 | [-39, -9] | 7.8 | -3.14 | 6147.9 | 0.002^**^ |
| PREC: i × SNR | -1.6 | [-7, 4] | 3.0 | -0.59 | 187.2 | 0.60 |
| CURR: I × SNR | -7.4 | [-18, 5] | 5.6 | -1.31 | 136.0 | 0.19 |
| PREC: i × CURR: I × SNR | 0.2 | [-10, 10] | 4.7 | 0.05 | 6150.0 | 0.96 |

*Note*. ^**^ *p* < 0.01, ^***^ *p* < 0.001. Estimates are given in ms. Trials with word recognition errors are excluded from analyses. The reported model is ONS = PREC × CURR × SNR + (1 + PREC + CURR | SUB). Trial type was dummy coded with congruent trials as the reference level and SNR was mean centered; the intercept represents cC trials at +6 dB SNR. PREC: i = preceding incongruent trial; CURR: I = current incongruent trial. The variances for the by-subjects random effects were 10858 for the intercept, 230 for preceding trial, and 4667 for current trial with an adjusted intraclass correlation coefficient of 0.360.

**Supplementary Results**

**Log-Transformed SOTs**

Due to a slight positive skew of the residuals for models of SOTs, we also ran the models after log-transforming SOTs. The inference of fixed effects was comparable after the log transformation for the analysis of congruency sequence effects and the analysis of SNR effects, as reported below.

***Analysis of Congruency Sequence Effects for Log-Transformed SOTs***

The model that converged was SOT = PREC × CURR × SNR + (1 + CURR | SUB), where PREC is preceding trial type dummy coded with congruent as the reference level, CURR is current trial type dummy coded with congruent as the reference level, SNR is the mean-centered SNR, and SUB is the by-subjects random effects term. The estimates for the intercept (b = 6.09, SE = 0.03, t(59.78) = 162.80, p < .001), current trial incongruency (b = 0.16, SE = 0.02, t(89.46) = 8.11, p < .001), and the combined effect of current and preceding trial incongruency (b = -0.04, SE = 0.02, t(2906.48) = -2.00, p = 0.045) were all significant for log SOTs across +6 and +8 dB SNR conditions. The effect of current trial incongruency indicated that participants were significantly slower for cI than cC trials, demonstrating the expected conflict effect. Additionally, the significant interaction revealed that participants were significantly faster for iI than cI trials, reflecting post-conflict improvements in SOTs. The fixed effects of preceding trial incongruency (b = -0.02, SE =0.01, t(2895.02) = -1.25, p = 0.21), SNR (b = -0.03, SE = 0.04, t(59.78) = -0.67, p = 0.51), preceding trial incongruency × SNR (b = -0.01, SE = 0.01, t(2895.05) = -0.72, p = 0.47), current trial incongruency × SNR (b = -0.01, SE = 0.02, t(89.60) = -0.68, p = 0.50), and the three-way interaction (b = 0.02, SE = 0.02, t(2906.33) = 1.07, p = 0.29) were not significant. The observed pattern of statistical significance and direction of effects for log-transformed SOTs matched that of raw SOTs.

***Analysis of SNR Effects for Log-Transformed SOTs***

As for the raw SOTs, the best-fitting model was SOT = PREC × CURR + (1 + PREC × CURR | SUB), as the models with linear or quadratic SNR and their interactions did not improve model fit (*p*s > 0.22) when analyzing data combined across +4, +6, and +8 dB SNR conditions. The intercept, which reflected log SOT for cC trials (b = 6.10, SE = 0.03, t(88.75) = 213.44, p < .001), the effect of current trial incongruency (b = 0.17, SE = 0.02, t(87.72) = 9.00, p < .001), and the combined effect of preceding and current trial incongruency (b = -0.04, SE = 0.02, t(234.74) = -2.26, p = 0.02) were all significant. These effects indicated slower SOTs for cI than cC trials and faster SOTs for iI than cI trials. The fixed effect of preceding trial incongruency was not significant (b = -0.01, SE = 0.01, t(382.48) = -0.79, p = 0.43). The observed pattern of statistical significance and direction of effects for log-transformed SOTs matched that of raw SOTs.

**Temporal Dependencies**

To evaluate temporal dependencies in the data, we also ran analyses including normalized trial number as a fixed effect. The inferences for the fixed effects of interest were comparable when including normalized trial number for the analysis of congruency sequence effects and the analysis of SNR effects, as reported below.

***Analysis of Temporal Dependencies for Congruency Sequence Effects in Accuracy***

We first ran a logistic mixed-effects model predicting accuracy for the +6 and +8 dB SNR data with a four-way interaction between fixed effects and the random effects structure from the model that did not evaluate temporal dependencies: REC = PREC × CURR × SNR × TRIAL + (1 | SUB). This model failed to converge. Inspection of the output revealed that none of the interactions between trial number and SNR reached significance. We therefore ran a simplified model removing all interactions that included both trial number and SNR. The resulting model still failed to converge, and results showed that none of the interactions between trial number and preceding trial were significant. After removing interactions involving both trial number and preceding trial, the final model was as follows: REC = PREC × CURR × SNR + TRIAL + CURR:TRIAL + (1 | SUB).

Fixed effects for preceding trial, current trial, SNR, and their interactions were comparable to the original model without trial number. The intercept, reflecting estimated accuracy for cC trials at +7 dB SNR in the middle of the experiment, was significant (b = 2.48, SE = 0.11, z = 22.57, p < .001). A significant effect of current trial incongruency (b = -2.64, SE = 0.12, z = -22.06, p < .001) indicated reduced accuracy for cI relative to cC trials. The magnitude of the interaction between preceding and current trial type was similar to the model that did not include trial number, albeit slightly reduced (b = 0.37, SE = 0.21, z = 1.79, p = .07). There was also a similar trend toward an interaction between current trial type and SNR (b = 0.21378, SE = 0.12, z = 1.79, p = .07).

Regarding the effects of trial number, accuracy for cC trials significantly decreased with trial number (b = -0.26, SE = 0.09, z = -2.85, p = .004), while a significant interaction between current trial type and trial number indicated that accuracy for cI trials significantly increased with trial number (b = 0.47, SE = 0.10, z = 4.75, p < .001). Since interactions between trial number and preceding trial type were dropped from the final model as described above, this is suggestive of larger interference effects toward the beginning than the end of the task regardless of whether the preceding trial was congruent or incongruent (see Figure S1). That is, congruency sequence effects on word recognition accuracy were stable across trials.


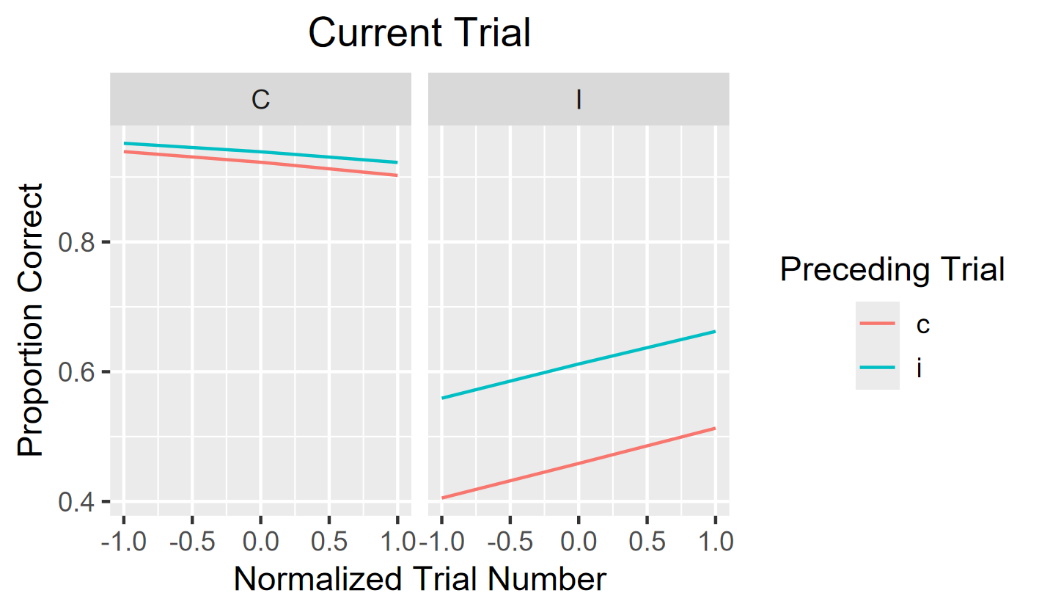


**Figure S1.** Estimated word recognition accuracy by normalized trial number and preceding trial type for current congruent trials (left panel) and current incongruent trials (right panel).

***Analysis of Temporal Dependencies for Congruency Sequence Effects in SOTs***

For the +6 and +8 dB SOT data, we conducted a linear mixed-effects model with a four-way interaction between fixed effects and the random effects structure from the model that did not evaluate temporal dependencies: SOT = PREC × CURR × SNR × TRIAL + (PREC + CURR | SUB). The magnitude of fixed effects was comparable to the original model that did not include trial number. Specifically, there were significant effects for the intercept (b = 473.20, SE = 14.68, t(59) = 32.24, p < .001), which represented SOTs for cC trials at +7 dB SNR in the middle of the experiment, as well as for current trial incongruency (b = 83.87, SE = 10.24, t(87) = 8.19, p < .001) and the preceding-by-current trial type interaction (b = -24.23, SE = 11.44, t(2877) = -2.12, p = .03). Consistent with the congruency sequence effects reported in the main text, cI trials were significantly slower than cC trials, but this interference effect was reduced when the preceding trial was incongruent.

However, effects of preceding and current trial type were modulated by trial number. This was reflected in a significant effect of trial number (b = -15.30, SE = 4.52, t(2828) = -3.39, p < .001) and significant interactions between preceding trial type and trial number (b = 17.18, SE = 7.30, t(2862) = 2.35, p = .02); current trial type and trial number (b = 17.18, SE = 751, t(2864) = 2.16, p = .03); and preceding trial type, current trial type, and trial number (b = -31.83, SE = 11.28, t(2857) = -2.82, p = .005). These effects emerged because participants became significantly faster on cC trials (b = -15.30, SE = 4.52, t(2834) = -3.38, p < .001) and iI trials (b = -13.74, SE = 6.21, t(2870) = -2.21, p = .03) with a +1 SD increase in trial number (about 43 trials). In contrast, SOTs for cI trials (b = 0.92, SE = 6.00, t(2871) = 0.15, p = .87) and iC trials (b = 1.88, SE = 5.73, t(2867) = 0.33, p = .74) did not significantly change with trial number. Thus, while iI trials were consistently faster than cI trials, this advantage for iI trials grew over the course of the experiment (see Figure S2, right panel). This suggests that participants became more efficient at deploying reactive cognitive control over the course of the speech recognition task.


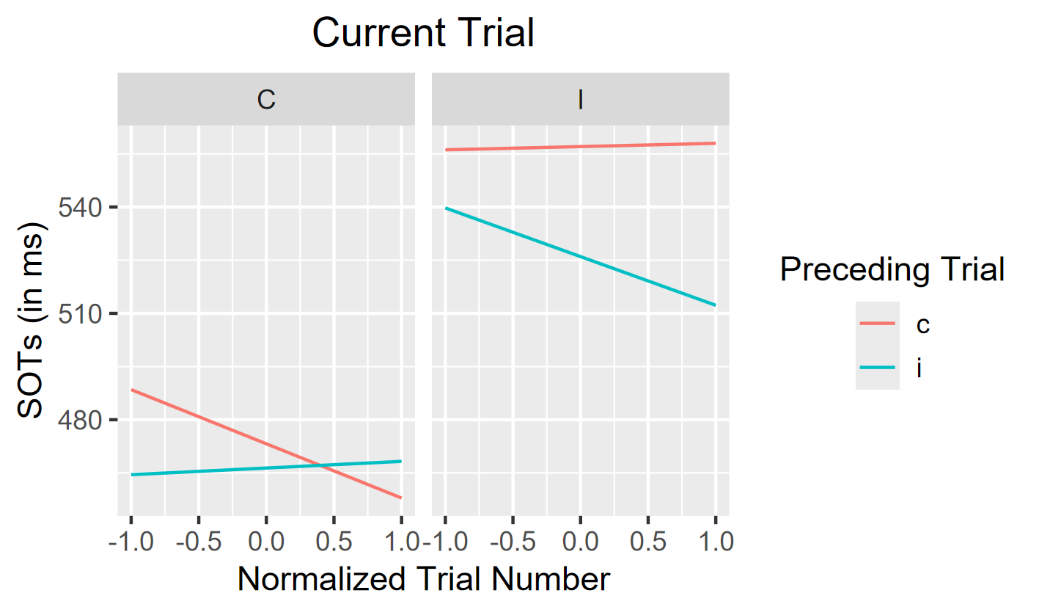


**Figure S2.** Estimated SOTs by normalized trial number and preceding trial type for current congruent trials (left) and current incongruent trials (right).

***Analysis of Temporal Dependencies for SNR Effects in Accuracy***

We first ran a logistic mixed-effects model predicting accuracy for the data including +4, +6, and +8 dB SNR with a four-way interaction between fixed effects and the random effects structure from the model that did not evaluate temporal dependencies: REC = PREC × CURR × SNR × TRIAL + (1 | SUB). This model failed to converge. Inspection of the output revealed that none of the interactions between trial number and SNR reached significance, so these were removed. The final model that converged was REC = PREC × CURR × SNR + TRIAL + PREC:TRIAL + CURR:TRIAL + PREC:CURR:TRIAL + (1 | SUB).

Fixed effects for preceding trial, current trial, SNR, and their interactions were comparable to the original model without trial number. These included a significant intercept (b = 2.63, SE = 0.09, z = 27.92, p < .001), which reflected accuracy for cC trials at +6 dB SNR in the middle of the experiment. There was also a significant effect of current trial incongruency (b = -2.89, SE = 0.10, z = -28.20, p < .001), indicating lower word recognition for cI than cC trials. The preceding by current trial type interaction was significant (b = 0.55, SE = 0.18, z = 3.09, p = .002), indicating higher iI than cI accuracy. Finally, a significant SNR effect (b = -0.14, SE = 0.06, z = -2.43, p = .02) coupled with the significant interaction between current trial incongruency and SNR (b = 0.24, SE = 0.06, z = 3.83, p < .001) revealed that the interference effect decreased with increasing SNR when the preceding trial was congruent, as cC performance declined and cI performance improved at higher SNRs.

With regards to trial number, recognition accuracy for cC trials significantly decreased over the course of the task (b = -0.26, SE = 0.09, z = -2.72, p = .007), while cI accuracy significantly improved (b = 0.39, SE = 0.10, z = 3.71, p < .001). None of the interactions between preceding trial and trial number were significant (*p*s > .35). Thus, interference was reduced over time due to falling accuracy on congruent trials and rising accuracy on incongruent trials, regardless of preceding trial type. As described above, interactions between trial number and SNR were removed from the model, suggesting that trial number effects on accuracy were consistent across SNRs.

***Analysis of Temporal Dependencies for SNR Effects in SOTs***

For the analysis of SNR effects including +4, +6, and +8 dB SNR, we first added a fixed effect of normalized trial number and its interactions to the final linear mixed-effects model of SNR effects in SOTs, which did not include a fixed effect of SNR: SOT = PREC × CURR × TRIAL + (PREC × CURR | SUB). This model did not converge, but inspection revealed a significant 3-way interaction between preceding trial type, current trial type, and normalized trial number. We therefore simplified the random-effects term until convergence was reached: SOT = PREC × CURR × TRIAL + (CURR | SUB).

Fixed effects corroborated those reported in the analysis that did not include trial number. The intercept (b = 471.85, SE = 11.54, t(96) = 40.88, p < .001) indicated SOTs for cC trials in the middle of the task and was significant. The fixed effect of current trial incongruency (b = 91.92, SE = 9.33, t(126) = 9.86, p < .001) revealed significantly slower responses for cI than cC trials, while a significant interaction between preceding and current trial type (b = -20.44, SE = 9.34, t(4383) = -2.19, p = .03) emerged due to faster responses on iI than cI trials.

Trial number modulated the effects of preceding and current trial type on SOTs. A significant effect of normalized trial number indicated that participants became faster on cC trials over the course of the task (b = -17.76, SE = 3.58, t(4347) = -4.96, p < .001). This decrease in SOTs with trial number was larger for cC trials than for iC trials, as reflected by a significant preceding trial-by-trial number interaction (b = 17.87, SE = 5.89, t(4355) = 3.03, p = .002), and was larger for cC trials than for cI trials, as reflected by a significant current trial-by-trial number interaction (b = 17.32, SE = 6.11, t(4379) = 2.83, p = .004). Finally, the three-way interaction between preceding trial, current trial, and normalized trial number was significant (b = -36.79, SE = 9.21, t(4366) = -4.00, p < .001), due to faster responding to iI trials over the course of the task. While participants’ responses became faster with a +1 SD increase in trial number for both cC (b = -17.76, SE = 3.58, t(4347) = -4.96, p < .001) and iI trials (b = -19.36, SE = 5.10, t(4390) = -3.80, p < .001), SOTs for iC (b = 0.11, SE = 4.68, t(4360) = 0.02, p = 0.98) and cI trials (b = -0.44, SE = 4.95, t(4391) = -0.09, p = 0.93) did not significantly change.
